# Insecticide resistance and resistance mechanisms in bed bugs, *Cimex* spp. (Hemiptera: Cimicidae)

**DOI:** 10.1186/s13071-017-2232-3

**Published:** 2017-06-29

**Authors:** Kai Dang, Stephen L. Doggett, G. Veera Singham, Chow-Yang Lee

**Affiliations:** 1 Urban Entomology Laboratory, Vector Control Research Unit, School of Biological Sciences, Universiti Sains Malaysia, 11800 Penang, Malaysia; 2 Department of Medical Entomology, NSW Health Pathology, Westmead Hospital, Westmead, NSW 2145 Australia; 3 Centre for Chemical Biology, Universiti Sains Malaysia, 10 Persiaran Bukit Jambul, 11900 Penang, Malaysia

**Keywords:** Bed bug, *Cimex lectularius*, *Cimex hemipterus*, Insecticide resistance, Mechanism, Molecular basis, Resistance monitoring

## Abstract

The worldwide resurgence of bed bugs [both *Cimex lectularius* L. and *Cimex hemipterus* (F.)] over the past two decades is believed in large part to be due to the development of insecticide resistance. The transcriptomic and genomic studies since 2010, as well as morphological, biochemical and behavioral studies, have helped insecticide resistance research on bed bugs. Multiple resistance mechanisms, including penetration resistance through thickening or remodelling of the cuticle, metabolic resistance by increased activities of detoxification enzymes (e.g. cytochrome P450 monooxygenases and esterases), and knockdown resistance by *kdr* mutations, have been experimentally identified as conferring insecticide resistance in bed bugs. Other candidate resistance mechanisms, including behavioral resistance, some types of physiological resistance (e.g. increasing activities of esterases by point mutations, glutathione S-transferase, target site insensitivity including altered AChEs, GABA receptor insensitivity and altered nAChRs), symbiont-mediated resistance and other potential, yet undiscovered mechanisms may exist. This article reviews recent studies of resistance mechanisms and the genes governing insecticide resistance, potential candidate resistance mechanisms, and methods of monitoring insecticide resistance in bed bugs. This article provides an insight into the knowledge essential for the development of both insecticide resistance management (IRM) and integrated pest management (IPM) strategies for successful bed bug management.

## Background

Chemical control remains the most important and widely used strategy against most insect pests around the world. However, studies have shown that multiple resistance mechanisms in insects confer resistance to a range of insecticide classes [1–7]. Recently, researchers have used new techniques and advances in genomic research (e.g. transcriptomic sequencing and whole-genome sequencing) to identify the mechanisms that govern insecticide resistance in bed bugs (Table 1). Previous bioassay, genetic, morphological, biochemical and behavioral studies, also have made significant progress in the understanding of bed bug insecticide resistance mechanisms such as penetration resistance, metabolic resistance and knockdown resistance. This review focuses on resistance to different insecticide classes and the underlying mechanisms in bed bugs. Other potential candidate resistance mechanisms in bed bugs are also reviewed.

**Table 1:** Progress in morphological, behavioral, biochemical, bioassay, and genetic characterization of insecticide resistance mechanisms in bed bugs (*Cimex* spp*.*)

Year	Characterization	Targets	Methods	Accession number^a^	Resistance mechanisms	Reference
*C. lectularius*						
2009	Behavioral	–	Bioassay: SC	–	Behavioral resistance	[89]
2009; 2015;2016	Bioassay	P450s; esterases	Bioassay (SC/T) plus synergists (e.g. PBO, PBH, EN16/5–1)		Metabolic resistance: P450s, esterases	[127, 128, 136]
2008; 2011; 2016	Biochemical	P450s; GSTs; esterases	Biochemical assays	–	Metabolic resistance: P450s, GSTs and esterases	[64, 113, 156]
2016	Morphological	Cuticle	SEM	–	Penetration resistance	[95]
2008	Genetic	VGSC	Cloning and sequencing (RACE)	FJ031996; FJ031997	Target site insensitivity: *kdr*	[156]
2011	Genetic	Transcriptome	454 pyrosequencing (Roche 454 GS FLX Titanium platform)	SRA024509	Metabolic resistance: P450s	[104]
2011	Genetic	Transcriptome	High-throughput sequencing (Roche 454 Titanium platform)	SRA043735	Metabolic resistance: P450s, GSTs and esterases; Target site insensitivity: *kdr*	[113]
2012	Genetic	RNA-seq	Illumina high-throughput sequencing (GAII platform)	GSE31823	Metabolic resistance: P450s, GSTs, ABC-transporters, esterases; Penetration resistance; Target site insensitivity: *kdr*	[92]
2012	Genetic	ClCPR	Cloning and sequencing (RACE)	JQ178363	Metabolic resistance: P450s	[122]
2012	Genetic	ClAChE1; ClAChE2; ClSChE	Cloning and sequencing (RACE)	JN563927; GU597837;GU597838;GU597839	–	[168]
2013	Genetic	CPRR	Data from NCBI	–	Penetration resistance	[94]
2013	Genetic	Transcriptome	454 pyrosequencing (Roche 454 GS FLX Titanium platform)	–	Metabolic resistance: P450s, esterases, ABC-transporters; Penetration resistance; Target site insensitivity: *kdr*	[90]
2016	Genetic	Genome	Illumina high-throughput sequencing (Illumina HiSeq2000s)	SRS580017	Metabolic resistance: P450s, esterases, ABC-transporters, GSTs; Penetration resistance; Target site insensitivity: *kdr*	[197]
2016	Genetic	Genome; RNA-seq	Illumina high-throughput sequencing	SRS749263; SRR1790655	Target site insensitivity: *kdr*, putative GABA receptors; Metabolic resistance:P450s, GSTs, esterases	[93]
*C. hemipterus*						
2011	Bioassay	P450s	Bioassay (SC) plus PBO	–	Metabolic resistance: P450s	[129]
2007	Biochemical	P450s; GSTs; Esterases	Biochemical assays	–	Metabolic resistance: GSTs, and esterases	[56]
2015	Genetic	VGSC(Part)	Sanger sequencing	–	Target site insensitivity: *kdr*	[17]

*Abbreviations*: *EN16/5–1* 6-[2-(2-butoxyethoxy) ethoxymethyl]-5-propyl-2, 3-dihydrobenzofuranby [127], *PBH* 3-Phenoxybenzyl hexanoate, a surrogate substrate for carboxylesterases and oxidases [136], *SC* surface contact, *T* topical application, *SEM* scanning electron microscope, *ClCPR Cimex lectularius* NADPH-cytochrome P450 reductase [122], *CPRR* cuticular protein with the rebers and riddiford consensus [94], *ClAChE1*, *ClAChE2* two *C. lectularius* acetylcholinesterases, *ClSChE C. lectularius* salivary gland-specific cholinesterase-like protein [168], *RACE* rapid amplification of cDNA ends, *ABC-transporters* **A**TP-**b**inding **c**assette (ABC) transporters

^a^Data from GenBank at NCBI (National Center for Biotechnology Information)

### Bed bugs

The common bed bug *Cimex lectularius* L. and the tropical bed bug *C. hemipterus* (F.) (Hemiptera: Cimicidae) are two cryptic and nocturnal ectoparasites that have adapted to feed on human blood [8, 9]. *Cimex lectularius* is most prevalent in temperate regions, whereas *C. hemipterus* is found mainly in tropical and subtropical regions [8, 10]. However, there is overlap in the regions where both species can be found, such as Thailand [11, 12], Africa [13, 14], Australia [15–17] and more recently in Florida, USA [18]. Approximately 70% of people who are bitten by *C. lectularius* experience allergic reactions ranging from mild to severe, including itchiness, erythematous rash, or urticaria [19–22], although fewer people react on initial exposure. For *C. hemipterus*, the percentage of the population that produces a clinical reaction is unknown, with described skin reactions including the formation of papular lesions with associated itch, which resolve around 1 h after the bite [23]. In addition, scratching may lead to secondary bacterial infections such as cellulitis, impetigo, ecthyma, and lymphangites [10, 21]. Only a small number of people may have no visible effect after repeated bed bug bites, and this lack of response may depend on previous exposure, although some people never develop a reaction despite multiple bites over time [24, 25]. Controlled laboratory studies have shown that bed bugs are capable of transmitting *Trypanosoma cruzi* (the etiological agent of Chagas disease) and *Bartonella quintana* (the etiological agent of trench fever) [26, 27]. However, to date there is no evidence to support the premise that bed bugs transmit these or other pathogens to humans in their natural habitat [10, 19, 21, 27].

Bed bugs have a long association with humans and were widespread and common world wide before World War II [9]. Soon thereafter, modern insecticides such as the organochlorine dichloro-diphenyl trichloroethane (DDT) were discovered and became a fast and an inexpensive method to control insect pests, including bed bugs. As a consequence, bed bugs gradually became uncommon, especially in economically developed countries, in the latter half of the twentieth century [9, 21, 28]. Unfortunately, over the last 15–20 years bed bugs have made a resurgence around the world with multiple reports of their comeback in the published literature, and popular main stream and social media (Table 2). Recent bed bug infestations have been reported from hotels, motels, homes and apartment complexes, cinemas, offices, retail outlets, public transportation, commercial flights, schools, and healthcare facilities (including neonatal units) [21, 28, 29]. Several factors, such as an increase in local and international travel, frequent exchange of second-hand items, poor pest management and insecticide resistance, have been suspected to be amongst the factors contributing to the global resurgence of bed bugs. Nevertheless, insecticide resistance has largely been incriminated as the main reason for the comeback of these nuisance pests [30].

**Table 2:** Reports of bed bug (*Cimex* spp.) resurgence from around the world since the beginning of the 21th century

Continent	Country	Species	Reference
Asia	One of the Arabian Gulf States	*Cimex* spp.^a^	[205]
	Bangladesh	*C. hemipterus*	[206]
	China (Mainland)	*C. lectularius*, *C. hemipterus*	[207, 208]
	Taiwan	*C. hemipterus*	[209]
	India	*C. lectularius*, *C. hemipterus*	[29, 205]
	Iran	*C. lectularius*	[210]
	Israel	*C. lectularius*, *C. hemipterus*	[211, 212]
	Japan	*C. lectularius*	[213]
	Kuwait	*C. lectularius*	[214]
	Malaysia	*C. hemipterus*	[129, 215–217]
	Pakistan	*C. lectularius*	[218]
	Singapore	*C. hemipterus*	[215]
	South Korea	*C. lectularius*	[219]
	Sri Lanka	*C. hemipterus*	[56]
	Thailand	*C. lectularius*, *C. hemipterus*	[12, 220]
Africa	Ethiopia	*Cimex* spp.^a^	[221]
	Kenya	*C. hemipterus*	[17]
	Nigeria	*C. lectularius*, *C. hemipterus*	[222, 223]
	Rwanda	*C. hemipterus*	[224]
	Sierra Leone	*C. lectularius*, *C. hemipterus*	[14]
	South Africa	*C. lectularius*, *C. hemipterus*	[13]
	Tanzania	*C. lectularius*, *C. hemipterus*	[55, 225]
	Uganda	*Cimex* spp.^a^	[226]
Americas	Argentina	*C. lectularius*	[227]
	Brazil	*C. lectularius*, *C. hemipterus*	[228–230]
	Canada	*C. lectularius*	[231, 232]
	Chile	*C. lectularius*	[227]
	Colombia	*C. lectularius*	[233]
	Cuba	*C. hemipterus*	[209]
	Mexico	*Cimex* spp.^a^	[28]
	Panama	*C. hemipterus*	[209]
	Peru	*C. lectularius*	[234]
	USA	*C. lectularius*, *C. hemipterus*	[18, 48, 157, 209, 235, 236]
	Venezuela	*C. lectularius*, *C. hemipterus*	[209, 237]
Europe	Austria	*C. lectularius*	[238]
	Czech Republic	*C. lectularius*	[205, 238]
	Denmark	*C. lectularius*	[239]
	France	*C. lectularius*	[158, 240, 241]
	Germany	*C. lectularius*	[159, 242–244]
	Hungary	*C. lectularius*	[245]
	Italy	*C. lectularius*	[246, 247]
	Norway	*C. lectularius*	[238]
	Poland	*C. lectularius*	[238]
	Spain	*C. lectularius*	[238]
	Russia	*C. lectularius*	[248, 249]
	Sweden	*C. lectularius*	[238]
	Slovakia	*C. lectularius*	[238]
	Switzerland	*C. lectularius*	[238, 250]
	UK	*C. lectularius*	[205, 251]
Oceania	Australia	*C. lectularius*, *C. hemipterus*	[15–17, 54, 252, 253]
	New Zealand	*C. lectularius*	[254]

^a^ *Cimex* spp., no indication of the species identification in the report

### Insecticide resistance in bed bugs

Insecticide resistance is defined by the Insecticide Resistance Action Committee (IRAC) [31] as ‘a heritable change in the sensitivity of a pest population that is reflected in the repeated failure of a product to achieve the expected level of control when used according to the label recommendation for that pest species’. In other words, it is an inherited ability of a population to survive a lethal concentration of an insecticide product that would normally kill a wild population. This is due to alleles that confer appropriate resistance factors, which subsequently increase in frequency in response to insecticide selection pressure. However, insecticide resistance is not to be confused with insecticide tolerance. Unlike insecticide resistance, the latter is the natural ability to withstand insecticide action, and is not the result of genetic changes caused by the insecticide selection pressure [32].

### DDT resistance

DDT was a long-lasting, relatively inexpensive and unrestricted chemical that was used worldwide as an insecticide to control disease-carrying mosquitoes, flies, and lice during and after the World War II [33]. Beginning in 1942, DDT was heavily used to control bed bug infestations in military barracks [9]. The first case of control failure of DDT against *C. lectularius* was reported in 1947 from the barracks of the Naval Receiving Station in Pearl Harbor, Hawaii [34]. It is noteworthy that bed bugs may have started developing resistance to DDT within 5 years after the product was first used, and the rapid pace of resistance was probably due to the excessive and continuous use of the insecticide. By the 1950s, bed bug resistance to DDT was widespread (Table 3) [35–40]. During this period, cross-resistance to pyrethrins was also observed in both *C. lectularius* (from Israel) and *C. hemipterus* (from Tanzania) [35]. Although there is abundant literature demonstrating that bed bugs had developed marked resistance to DDT, bed bug infestations decreased dramatically and were effectively reduced to very low levels from the 1950s to the late 1970s in many developed countries. The repetitive and widespread use of DDT and subsequent insecticides (e.g. malathion, chlorpyrifos, and propoxur) had led to the significant decrease of bed bug infestations worldwide [9]. However, bed bug infestations were still a major problem in some developing countries, such as Sierra Leone [14], South Africa and rural Africa [13, 41, 42], as well as problematic in the poultry industry in many countries [8, 28, 43, 44].

**Table 3:** Reports of resistance to chlorinated hydrocarbons in bed bugs (*Cimex* spp.) by the 1970s

Species	Year	Chlorinated hydrocarbon	Location	Reference
*C. lectularius*	1947	DDT	USA (Hawaii)	[34]
	1949	DDT	Greece	[255–257]
	1952	DDT	USA (Ohio, Illinois, Indiana, Utah)	[36]
	1953	DDT	Belgian Congo	[258]
	1953	DDT	Israel	[259, 260]
	1953	DDT	Japan, Italy	[36]
	1954	HCH, dieldrin	Italy	[36]
	1955	DDT	USA (Colorado, Pennsylvania, Texas)	[36]
	1956	HCH, dieldrin	Israel	[36]
	1956	DDT	French Guiana	[261]
	1956	DDT	Iran	[262]
	1957	γ-HCH	Israel	[263]
	1957	DDT	Trinidad, Turkey	[36]
	1957	DDT, chlordane, dieldrin	Italy	[264]
	1958	Dieldrin, γ-HCH, aldrin, endrin, isodrin, α-chlordane, β-chlordane, methoxychlor, perthane, prolan	Israel	[35]
	1958	DDT	Lebanon	[265]
	1958	DDT	Japan, Korea, USA (Ohio, and two US naval vessels)	[266]
	1959	DDT	Hungary, Poland	[36, 40, 267]
	1960	HCH, dieldrin	Indonesia, Zambia, Rhodesia, Borneo	[37]
	1960	DDT	Borneo, Indonesia, Colombia	[36, 40]
	1960	DDT	Zimbabwe	[268]
	1961	DDT, HCH, dieldrin	South India	[36]
	1962	γ-HCH	India	[269]
	1962	DDT, HCH, dieldrin	South Africa	[37, 270]
	1963	DDT dieldrin	Gaza	[271]
	1967	DDT, HCH, dieldrin	Egypt	[37, 272]
	1971	γ-HCH	Zambia, Italy, Borneo	[40]
	1972	DDT	Papua-New Guinea	[273]
	1976	DDT, dieldrin	Almost everywhere	[38]
*C. hemipterus*	1955	DDT	West India	[36]
	1956	HCH, dieldrin	West India	[36]
	1956	DDT	Taiwan	[274]
	1956	DDT	India (Bombay State)	[275]
	1957	HCH, dieldrin	Tanzania, Kenya, Upper Volta	[37]
	1957	DDT	Hong Kong, Singapore	[264]
	1957	DDT	Kenya	[36]
	1957	Dieldrin	Ivory Coast	[35]
	1958	Dieldrin	Tanganyika	[276]
	1958	DDT	Mombasa, Somalia, Gambia, Hong Kong	[35]
	1958	Dieldrin	Mombasa, Gambia	[35]
	1958	γ-HCH	Mombasa, Somalia, Gambia	[35]
	1958	Methoxychlor	Mombasa, Somalia	[35]
	1959	DDT	Poland	[267]
	1959	HCH, dieldrin	Dahomeh, Zanzibar	[36, 37]
	1960	DDT	Malaysia, Thailand	[36, 37]
	1960	HCH, dieldrin	Malaysia	[36, 37]
	1961	DDT, HCH, dieldrin	Madagascar, South India	[36, 37]
	1961	DDT	Tanzania (Zanzibar)	[277]
	1962	Dieldrin	Tanzania (Magugu)	[278]
	1970	DDT	Papua-New Guinea	[279]

*Abbreviations*: HCH hexachlorocyclohexane, γ-HCH *gamma*-hexachlorocyclohexane, also known as lindane, gammaxene, gammallin and sometimes incorrectly called benzene hexachloride (BHC)

### Pyrethroid resistance

Pyrethroids, the synthetic analogues of pyrethrin in pyrethrum, an extract of *Chrysanthemum cinerariaefolium* flower, are a class of highly effective and extremely efficient neurotoxic insecticides [45, 46]. However, with the worldwide resurgence of bed bugs over the last two decades, resistance to pyrethroids (Table 4) has been documented in many parts of the world for both *C. lectularius* [16, 30, 47–54] and *C. hemipterus* [12, 16, 17, 55, 56].

**Table 4:** Published reports of insecticide resistance and product efficacy in modern bed bugs (*Cimex* spp.), post 2000

Year	Insecticide	Method	Location/Strain	Susceptibility/efficacy	Resistance ratio	Reference
*C. lectularius*						
2006	α-cypermethrin^a^	SC	UK (3 field strains)	Resistant	–	[47]
2006	Bendiocarb^b^	SC	UK (3 field strains)	Resistant	–	[47]
2006	Deltamethrin^a^	SC	USA (Arlington, VA)	Resistant	>300	[48]
2006	Chlorfenapyr^g^	SC	Susceptible Harlan strain	Less effective		[48]
2007	Deltamethrin^a^	SC	USA: Cincinnati, OH(CIN1, CIN2, CIN3); Lexington, KY (LEX1)	Resistant	>12,765	[30]
2007	λ-cyhalothrin^a^	SC	USA (Cincinnati, OH[CIN1])	Resistant	>6123	[30]
2007	Deltamethrin^a^	SC	USA (Los Angeles, CA [LA2]; Kissimmee, FL[KIS1]; Vienna, VA[VIN1])	Resistant	–	[30]
2007	Deltamethrin^a^	SC	USA (Los Angeles, CA [LA1])	Susceptible		[30]
2008	Deltamethrin^a^	SC	USA (New York City, NY [NY-BB])	Resistant	>250	[156]
2008	Bifenthrin^a^	SC	USA (Arkansas: Washington, Carroll, Lafayette)	Susceptible		[44]
2008	λ-cyhalothrin^a^	SC	USA (Arkansas: Washington, Carroll, Lafayette)	Susceptible		[44]
2008	Permethrin^a^	SC	USA (Arkansas: Washington, Carroll, Lafayette)	Susceptible		[44]
2008	Carbaryl^b^	SC	USA (Arkansas: Washington, Carroll, Lafayette)	Susceptible		[44]
2008	Imidacloprid^c^	SC	USA (Arkansas: Washington, Carroll, Lafayette)	Susceptible		[44]
2008	Fipronil^d^	SC	USA (Arkansas: Washington, Carroll, Lafayette)	Susceptible		[44]
2008	Diazinon^e^	SC	USA (Arkansas: Washington, Lafayette)	Susceptible		[44]
2008	Diazinon^e^	SC	USA (Arkansas: Carroll)	Resistant	–	[44]
2008	Dichlorvos^e^	SC	USA (Arkansas: Washington, Carroll, Lafayette)	Resistant	–	[44]
2008	Spinosad^f^	SC	USA (Arkansas: Washington, Carroll, Lafayette)	Resistant	–	[44]
2008	Chlorfenapyr^g^	SC	USA (Arkansas: Washington, Carroll, Lafayette)	Less effective		[44]
2008	DDT^h^	SC	USA (Arkansas: Washington, Carroll, Lafayette)	Resistant	–	[44]
2008	Chlorfenapyr^g^	SC	USA (Cincinnati, OH)	Less effective		[69]
2009	Pirimphos-methyl^e^	T	Australia (Sydney strain)	Susceptible	2.6	[49, 52]
2009	Imidacloprid^c^	T	Australia (Sydney strain)	Susceptible	2.6	[49, 52]
2009	Bendiocarb^b^	T	Australia (Sydney strain)	Resistant	250	[49, 52]
2009	Deltamethrin^a^	T	Australia (Sydney strain)	Resistant	370,000	[49, 52]
2009	Permethrin^a^	T	Australia (Sydney strain)	Resistant	1,235,000	[49, 52]
2009	Diazinon^e^	T/SC	Australia (Sydney strain)	Effective		[50, 51]
2009	Pyrethrins^a^	T/SC	Australia (Sydney strain)	Resistant	–	[50, 51]
2009	β-cyfluthrin^a^	T/SC	Australia (Sydney strain)	Resistant	–	[50, 51]
2009	Tetramethrin^a^	T/SC	Australia (Sydney strain)	Resistant	–	[50, 51]
2009	Deltamethrin^a^	SC	USA (Cincinnati, OH [CIN-1])	Resistant	>2588	[128]
2009	Deltamethrin^a^	SC	USA (Worcester, MA[WOR-1])	Resistant	>2588	[128]
2010	Deltamethrin^a^	SC	USA (New York City, NY)	Resistant	>9375	[162]
2010	λ-cyhalothrin^a^	SC	USA (New York City, NY)	Resistant	6990	[162]
2010	Chlorfenapyr^g^	SC	USA (Cincinnati, OH [CIN-1])	Effective	–	[65]
2010	Chlorfenapyr^g^	SC	USA (Worcester, MA[WOR-1])	Effective	–	[65]
2010	Phenothrin^a^	SC/T	Japan (four field strains)	Resistant	–	[280]
2010	Permetrhin^a^	SC/T	Japan (four field strains)	Resistant	–	[280]
2010	Dichlorvos^e^	SC/T	Japan (four field strains)	Susceptible		[280]
2010	Fenitrothion^e^	SC/T	Japan (four field strains)	Susceptible		[280]
2010	Propoxur^b^	SC/T	Japan (four field strains)	Susceptible		[280]
2010	Deltamethrin^a^	SC	USA (Cincinnati, OH; Lexington, KY; Troy, MI; Dover, NJ; Frankfort, KY; Kalamazoo, MI; Worcester, MA; Smithtown, Plainview, New York, NY)	Resistant	–	[157]
2011	Deltamethrin^a^	IT	USA (Richmond, VA)	Resistant	5167	[113]
2011	β-cyfuthrin^a^	IT	USA (Richmond, VA)	Resistant	111	[113]
2011	Deltamethrin^a^	SC	USA (Richmond, VA)	Resistant	390.5	[281]
2011	Deltamethrin^a^	SC	USA (Cincinnati, OH)	Resistant	>340	[281]
2011	Deltamethrin^a^	SC	USA (Arlington, VA: Kramer)	Resistant	339.6	[282]
2011	Permethrin^a^	SC	USA (Arlington, VA: Kramer)	Resistant	>115.1	[282]
2011	Deltamethrin^a^	SC	USA (Richmond, VA)	Resistant	390.5	[282]
2011	Permethrin^a^	SC	USA (Richmond, VA)	Resistant	>291.7	[282]
2011	DDT^h^	SC	Thailand (Chiang Mai)	Resistant	–	[12]
2011	Dieldrin^h^	SC	Thailand (Chiang Mai)	Resistant	–	[12]
2011	Bendiocarb^b^	SC	Thailand (Chiang Mai)	Resistant	–	[12]
2011	Propoxur^b^	SC	Thailand (Chiang Mai)	Resistant	–	[12]
2011	Malathion^e^	SC	Thailand (Chiang Mai)	Resistant	–	[12]
2011	Fenitrothion^e^	SC	Thailand (Chiang Mai)	Resistant	–	[12]
2011	Cyfluthrin^a^	SC	Thailand (Chiang Mai)	Resistant	–	[12]
2011	Deltamethrin^a^	SC	Thailand (Chiang Mai)	Resistant	–	[12]
2011	Permethrin^a^	SC	Thailand (Chiang Mai)	Resistant	–	[12]
2011	β-cyhalothrin^a^	SC	Thailand (Chiang Mai)	Resistant	–	[12]
2011	Etofenprox^a^	SC	Thailand (Chiang Mai)	Resistant	–	[12]
2011	Diazinon^e^	Spray	Thailand (Chiang Mai)	Less effective	–	[12]
2011	Fenobucarb^b^	Spray	Thailand (Chiang Mai)	Less effective	–	[12]
2011	Esfenvalerate^a^	Spray	Thailand (Chiang Mai)	Less effective	–	[12]
2011	Cypermethrin^a^	Spray	Thailand (Chiang Mai)	Less effective	–	[12]
2011	Bifenthrin^a^	Spray	Thailand (Chiang Mai)	Less effective	–	[12]
2011	Chlorfenapyr^g^	Spray	Thailand (Chiang Mai)	Less effective	–	[12]
2011	Fipronil^d^	Spray	Thailand (Chiang Mai)	Less effective	–	[12]
2011	Imidacloprid^c^	Spray	Thailand (Chiang Mai)	Efficient	–	[12]
2011	Permethrin^a^	T, SC	Denmark	Resistant	–	[53]
2011	Deltamethrin^a^	SC	Denmark	Resistant	–	[53]
2011	Chlorpyrifos^e^	T, SC	Denmark	Effective	–	[53]
2012	Deltamethrin^a^	SC	USA (Columbus, OH)	Resistant	–	[92]
2012	Pyrethrins^a^	T	USA(New Haven, CT)	Resistant	–	[283]
2012	Cyfluthrin^a^	T	USA(New Haven, CT)	Resistant	–	[283]
2012	λ-cyhalothrin^a^	T	USA(New Haven, CT)	Resistant	–	[283]
2012	cis-cypermethrin^a^	T	USA(New Haven, CT)	Resistant	–	[283]
2012	Deltamethrin^a^	T	USA(New Haven, CT)	Resistant	–	[283]
2012	Deltamethrin^a^	T	USA (Cincinnati, OH [CIN-1], Plainview, NY [NY-1])	Resistant	–	[122]
2012	Neopynamine^a^	SC	France (Paris)	Resistant	–	[158]
2012	Sumithrin^a^	SC	France (Paris)	Resistant	–	[158]
2013	Deltamethrin^a^	SC	USA (CIN-1)	Resistant	51	[90]
2013	Deltamethrin^a^	SC	USA (CIN-1 S)	Resistant	32,700,000	[90]
2013	Deltamethrin^a^	SC	USA (NY-1)	Resistant	>300	[90]
2013	Deltamethrin^a^	T	USA (Richmond, VA)	Resistant	>200,000	[94]
2013	β-cyfuthrin^a^	T	USA (Richmond, VA)	Resistant	>160,000	[94]
2014	Deltamethrin^a^	SC	Germany (Berlin)	Resistant	3.8–5.1	[159]
2014	Deltamethrin^a^	SC	USA: New York (Brooklyn)	Susceptible	–	[284]
2015	Imidacloprid^c^/β-cyfluthrin^c^	SC	USA (Richmond and Epic Center strains)	Resistant	E: 3–5; FI: 121–493	[285]
2015	Acetamiprid^c^/bifenthrin^a^	SC	USA (Richmond and Epic Center strains)	Resistant	E: 39–1080; FI: 99–1943	[285]
2015	Deltamethrin^a^	SC	USA (Epic Center strain)	Resistant	392	[285]
2015	d-allethrin^a^	SC	Australia [NSW: Sydney (Abbotsford, Darlinghurst, North Parramatta, Northbridge, Redfern), Newcastle (Maryland); VIC: Melbourne (Ripponlea, South Yarra, Moonee Ponds), West Melbourne; WA: Perth (Cottesloe); NT: Alice springs; SA: Adelaide (Semaphore Park)]	Resistant	–	[16, 54]
2016	Imidacloprid^c^	T	USA (Jersey City, NJ)	Susceptible	2.0	[64]
2016	Imidacloprid^c^	T	USA (Troy, MI)	Resistant	462.6	[64]
2016	Imidacloprid^c^	T	USA (Cincinnati, OH)	Resistant	163.3	[64]
2016	Acetamiprid^c^	T	USA (Jersey City, NJ)	Resistant	31.7	[64]
2016	Acetamiprid^c^	T	USA (Troy, MI)	Resistant	>33,333	[64]
2016	Acetamiprid^c^	T	USA (Cincinnati, OH)	Resistant	>33,333	[64]
2016	Thiamethoxam^c^	T	USA (Jersey City, NJ)	Susceptible	2.4	[64]
2016	Thiamethoxam^c^	T	USA (Troy, MI)	Resistant	546	[64]
2016	Thiamethoxam^c^	T	USA (Cincinnati, OH)	Resistant	226.2	[64]
2016	Dinotefuran^c^	T	USA (Jersey City, NJ)	Resistant	46.8	[64]
2016	Dinotefuran^c^	T	USA (Troy, MI)	Resistant	198	[64]
2016	Dinotefuran^c^	T	USA (Cincinnati, OH)	Resistant	358.6	[64]
2016	Deltamethrin^a^	T	Australia: Parramatta(NSW), Alice Springs(NT) and Melbourne(VIC)	Resistant	–	[127]
2016	Deltamethrin^a^	SC	Australia (Parramatta[NSW])	Resistant	–	[95]
*C. hemipterus*						
2002	α-cypermethrin^a^	SC	Tanzania	Resistant	–	[55]
2002	Permethrin^a^	SC	Tanzania	Resistant	–	[55]
2007	DDT^h^	SC	Sri Lanka	Resistant	–	[56]
2007	Malathion^e^	SC	Sri Lanka	Resistant	–	[56]
2007	Propoxur^b^	SC	Sri Lanka	Resistant	–	[56]
2007	Deltamethrin^e^	SC	Sri Lanka	Resistant	–	[56]
2007	Permethrin^e^	SC	Sri Lanka	Resistant	–	[56]
2011	λ-cyhalothrin^a^	SC	Malaysia (Kmelayu14); Singapore (Serangoon)	Effective	–	[129]
2011	Bifentrin^a^	SC	Malaysia (Kmelayu14); Singapore (Serangoon)	Effective	–	[129]
2011	Fenitrothion^e^	SC	Malaysia (Kmelayu14); Singapore (Serangoon)	Effective	–	[129]
2011	Fipronil^d^	SC	Malaysia (Kmelayu14); Singapore (Serangoon)	Effective	–	[129]
2011	Imidacloprid^c^	SC	Malaysia (Kmelayu14); Singapore (Serangoon)	Effective	–	[129]
2011	DDT^h^	SC	Malaysia (Kmelayu14); Singapore (Serangoon)	Resistant	–	[129]
2011	DDT^h^	SC	Thailand (Bangkok, Chonburi, Phuket, Krabi)	Resistant	–	[12]
2011	Dieldrin^h^	SC	Thailand (Bangkok, Chonburi, Phuket, Krabi)	Resistant	–	[12]
2011	Bendiocarb^b^	SC	Thailand (Bangkok, Chonburi, Phuket, Krabi)	Resistant	–	[12]
2011	Propoxur^b^	SC	Thailand (Bangkok, Chonburi, Phuket, Krabi)	Resistant	–	[12]
2011	Malathion^e^	SC	Thailand (Bangkok, Chonburi, Phuket, Krabi)	Resistant	–	[12]
2011	Fenitrothion^e^	SC	Thailand (Bangkok, Chonburi, Phuket, Krabi)	Resistant	–	[12]
2011	Cyfluthrin^a^	SC	Thailand (Bangkok, Chonburi, Phuket, Krabi)	Resistant	–	[12]
2011	Deltamethrin^a^	SC	Thailand (Bangkok, Chonburi, Phuket, Krabi)	Resistant	–	[12]
2011	Permethrin^a^	SC	Thailand (Bangkok, Chonburi, Phuket, Krabi)	Resistant	–	[12]
2011	λ-cyhalothrin^a^	SC	Thailand (Bangkok, Chonburi, Phuket, Krabi)	Resistant	–	[12]
2011	Etofenprox^a^	SC	Thailand (Bangkok, Chonburi, Phuket, Krabi)	Resistant	–	[12]
2011	Diazinon^e^	Spray	Thailand (Bangkok, Chonburi, Phuket, Krabi)	Less effective	–	[12]
2011	Fenobucarb^b^	Spray	Thailand (Bangkok, Chonburi, Phuket, Krabi)	Less effective	–	[12]
2011	Esfenvalerate^a^	Spray	Thailand (Bangkok, Chonburi, Phuket, Krabi)	Less effective	–	[12]
2011	Cypermethrin^a^	Spray	Thailand (Bangkok, Chonburi, Phuket, Krabi)	Less effective	–	[12]
2011	Bifenthrin^a^	Spray	Thailand (Bangkok, Chonburi, Phuket, Krabi)	Less effective	–	[12]
2011	Chlorfenapyr^g^	Spray	Thailand (Bangkok, Chonburi, Phuket, Krabi)	Less effective	–	[12]
2011	Fipronil^d^	Spray	Thailand (Bangkok, Chonburi, Phuket, Krabi)	Less effective	–	[12]
2011	Imidacloprid^c^	Spray	Thailand (Bangkok, Chonburi, Phuket, Krabi)	Effective	–	[12]
2015	d-allethrin^a^	SC	Australia (North Queensland)	Resistant	>130	[17]
2015	d-allethrin^a^	SC	Australia (Sydney, [NSW]: Auburn)	Resistant	37	[17]
2015	d-allethrin^a^	SC	Malaysia (Kuala Lumpur)	Resistant	>130	[17]
2015	d-allethrin^a^	SC	Thailand (Bangkok, Chiang Mai)	Resistant	>130	[17]
2015	d-allethrin^a^	SC	Africa (Kenya)	Resistant	30	[17]

*Abbreviation*: *SC* Surface contact, *T* Topical application, *IT* Injection topical application [112]

^a^Pyrethroids

^b^Carbamates

^c^Neonicotinoids

^d^Phenylpyrazoles

^e^OPs

^f^Spinosyn

^g^Pyrrole

^h^Chlorinated hydrocarbons

### Neonicotinoid resistance

Imidacloprid, a neonicotinoid was introduced into the market in 1991 [57]. The neonicotinoids are now widely used against a wide variety of chewing and sucking pests [58], including the bed bugs [59–63]. Over the last few years, neonicotinoids have been combined with pyrethroids in formulated products, such as Temprid® SC (beta-cyfluthrin + imidacloprid), Transport® Mikron (bifenthrin + acetamiprid) and Tandem® (lambda-cyhalothrin + thiamethoxam) [10, 60, 63], and with diatomaceous earth (e.g. Alpine Dust Insecticide, with dinotefuran) [64] for the control of bed bugs. However, in a recent study, *C. lectularius* collected from human dwellings in Cincinnati and Michigan, USA have shown moderate to high levels of tolerance/resistance to various neonicotinoids [64]. Romero & Anderson [64] reported that resistance to neonicotinoids may likely be conferred by the increased enzymatic activities found in these populations. These findings indicate that tolerance or even resistance to neonicotinoids is now present in field bed bug populations.

### Pyrrole efficacy

Besides the conventional insecticides (e.g. pyrethroids, OPs, and carbamates), newer insecticide such as chlorfenapyr from the pyrrole class have also been evaluated [65, 66]. Pyrroles are a class of pro-insecticides that are activated by cytochrome P450 monooxygenases (P450s) to its more active metabolite [67]. Unlike neurotoxic insecticides (e.g. pyrethroids), pyrroles are mitochondrial electron transport inhibitors (METI) which disrupt the conversion of adenosine diphosphate (ADP) to adenosine triphosphate (ATP) (oxidative phosphorylation) in mitochondria of cells. This process results in loss of energy production, which leads to cell dysfunction and subsequent death of the organism [67]. Due to their novel mode of action, pyrroles (e.g. chlorfenapyr) are currently registered in more than 19 countries for the control of various insect and mite pests [68], especially against pyrethroid-resistant insect pests, including bed bugs [65, 66, 69, 70]. Nevertheless, there are increasing number of reports of insects and mites becoming resistant or cross-resistant to pyrroles [71–74], mainly due to metabolic resistance (e.g. P450s and esterases) [72, 75].

Despite no cases of resistance to chlorfenapyr has been reported in the bed bug so far, this compound has shown varying performance against bed bugs. While chlorfenapyr (Phantom®) was extremely slow-acting in laboratory residual bioassays [48], direct application of the product on the walls of infested apartments resulted in a 61% reduction in bed bug numbers in just 3 days [76]. Romero et al. [65] reported that chlorfenapyr is a non-repellent insecticide with long residual activity against *C. lectularius* based on laboratory studies. On the contrary, other researchers have found poor performance of this product even with laboratory susceptible bed bugs [21, 66]. In a field evaluation [69], Phantom® was widely sprayed throughout 15 apartments monthly for 5 months. The authors only achieved control in 12 units despite including a range of non-chemical means, yet three units remained infested. It is not possible to determine if this lack of complete control was due to poor product efficacy (and possible resistance) or due to bed bugs being reintroduced.

### Resistance to other insecticides

Besides resistance to DDT, by the 1950s both bed bug species also had developed resistance to other chlorinated hydrocarbon compounds (OCs) [e.g. *gamma*-HCH (1957), methoxychlor (1958), dieldrin (1958), aldrin (1958), and endrin (1958)] (Table 3) [35], organophosphates (OPs) (e.g. malathion) (1971) [77], and carbaryl (carbamate) (1972) [78]. Following the resurgence in the new millennia, resistance to OCs [56], OPs [12, 53, 56] and carbamates [12, 44, 49, 52] were also reported in bed bugs (Table 4). All these classes of insecticides have largely been banned for indoor use against bed bugs with exception to selected countries in Asia, Africa, and some countries in Latin America.

## Mechanisms of resistance

**Fig. 1:**
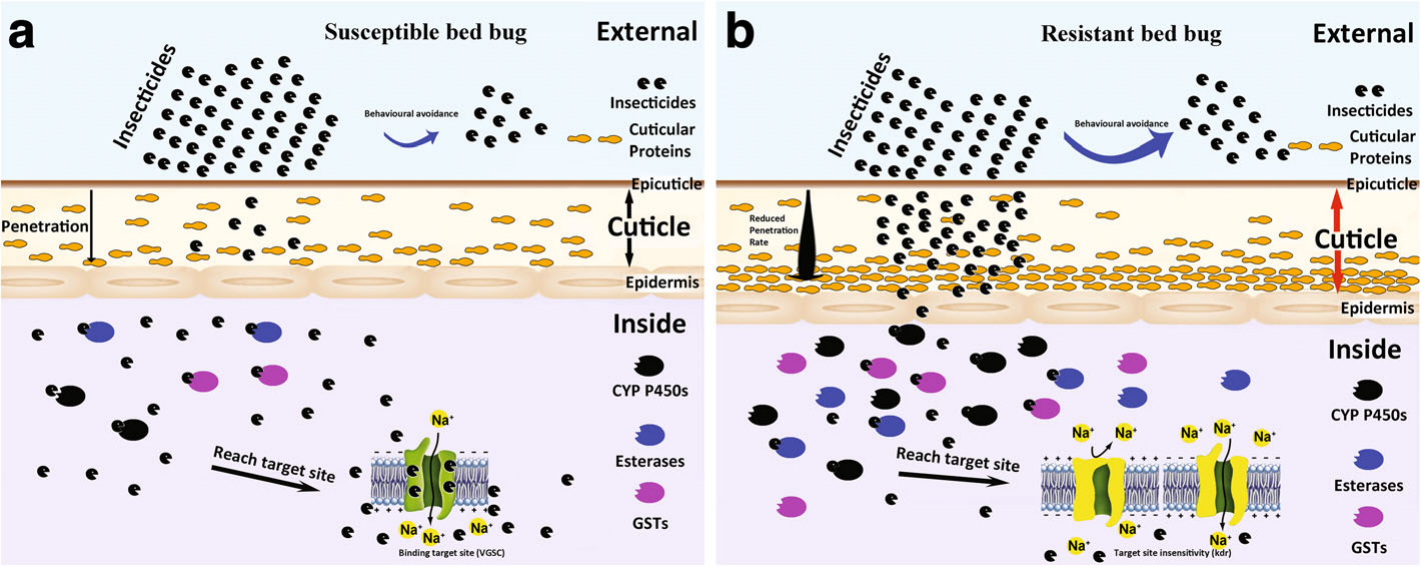
Schematic of potential behavioral and physiological changes involved in insecticide resistance in bed bugs. **a** Susceptible bed bug. **b** Resistant bed bug. The various forms of resistance act in compounding layers to counteract the effect of the insecticide. For example, direct application of an insecticide such as a pyrethroid may kill the bed bugs; however due to the ‘excito-repellency’ nature of this class of compounds, some bed bugs may avoid insecticide exposure (potential behavioral resistance). If the bed bugs come into contact with an insecticide, the cuticle may be thickened or remodelled by over-expression of cuticular proteins, which will reduce the rate of insecticide penetration (penetration resistance) beyond the cuticular layer. If the insecticides enter the insect, bed bugs can enhance metabolic detoxification (e.g. P450s, esterases, GSTs) to inhibit the insecticidal effect (metabolic resistance). Finally, if the insecticides reach the neurological system to act on the target sites (such as the VGSC), point mutations (e.g. *kdr* mutations) can reduce the sensitivity of the insecticide target site to the insecticide (target site insensitivity)

Insecticide resistance can be divided into two major types: behavioral resistance and physiological resistance [1, 79]. In behavioral resistance, the insect populations may develop the ability to avoid or reduce lethal insecticide exposure [79]. In contrast, physiological resistance refers to physiological modification mechanisms, including reduced cuticular penetration, increased metabolic detoxification, and decreased target site sensitivity. Behavioral and physiological resistance often coexist in insect pests [79, 80] and both forms could be involved with bed bug resistance (Table 5, Fig. 1). Herein the resistance mechanisms that have been reported in the bed bug are discussed, as well as candidate resistance mechanisms that are yet to be confirmed. So far, only penetration resistance, metabolic resistance (namely P450 and esterase), and target site insensitivity (namely *kdr*-type) have been found to confer resistance in bed bugs. Other resistance mechanisms that are yet to be detected in bed bugs are behavioral resistance, glutathione S-transferase (GST), altered acetylcholinesterase (AChE), insensitive γ-aminobutyric acid (GABA) receptor, altered nAChRs, and symbiont-mediated insecticide resistance. The role of ATP-binding cassette (ABC) transporters in upregulation of toxicant removal from the target site will be discussed. For the ease of the flow of discussion, we shall discuss according to types of the resistance mechanisms (behavioural, metabolic, target site insensitivity, etc.) and will highlight resistance mechanisms that are yet to be detected in bed bugs as candidate mechanisms.

**Table 5:** Resistance mechanisms (verified and candidate mechanisms) in bed bugs (*Cimex* spp.)

Mechanism			*C. lectularius*	*C. hemipterus*	Insecticide
Behavioral resistance			Candidate mechanism	Candidate mechanism	Pyrethroids
Physiological resistance	Penetration resistance	Cuticle	Verified by morphological study [95, 103]	Candidate mechanism	A broad spectrum of insecticide classes
	Metabolic resistance	P450s	Verified by RNAi [90] and synergism studies [127]	Verified by synergism studies [129]	Pyrethroids, OCs, OPs, carbamates, neonicotinoids and pyrroles
		Esterases	Verified by synergism studies [127]	Candidate mechanism	Carbamates, OPs, pyrroles, neonicotinoids and pyrethroids
		GSTs	Candidate mechanism	Candidate mechanism	OCs, OPs and pyrethroids
		ABC-transporters	Verified by RNAi [90]	Candidate mechanism	Pyrethroids, OCs, OPs, carbamates, and neonicotinoids
	Target site insensitivity	*Kdr*	Verified by QS combined with FCVB [162]	Candidate mechanism	Pyrethroids and DDT
		Altered AChEs	Candidate mechanism	Candidate mechanism	OPs and carbamates
		Insensitive GABA receptors	Candidate mechanism	Candidate mechanism	Cyclodienes (OCs) and phenylpyrazoles
		Altered nAChRs	Candidate mechanism	Candidate mechanism	Neonicotinoids
Symbiont-mediated resistance			Candidate mechanism	Candidate mechanism	OPs

### Behavioral resistance (candidate resistance mechanism)

Behavioral resistance can be divided into two broad categories: (i) stimulus-dependent behaviors (e.g. irritability and repellency), and (ii) stimulus-independent behavior (e.g. exophily, outside resting) [1, 79, 81]. Stimulus-dependent behaviors require sensory stimulation of the insect to detect a toxin-treated surface before acquiring a lethal dose, by which an avoidance response is elicted by the toxicant [79]. Such behaviors have been observed in a number of insect pests. The best known example is bait aversion to glucose in the German cockroach, *Blattella germanica* (L.), which was selected through field exposure to commercial cockroach baits [82–85]. Conversely, stimulus-independent behavior refers to insect behavior that prevents them from exposure to the toxicant [1]. These behaviors do not require sensory stimulation by the toxicant for avoidance to happen [79]. One example is ‘exophily’, which refers to the changes of behavior in anopheline mosquitoes from their regular habit of biting indoor, to biting outdoors [86]. This behavior decreases the prevalence of indoor mosquitoes, and hence reduces the potential for mosquitoes from contacting indoor insecticide applications [87, 88].

Although behavioral resistance has been documented in many insect pest groups, it has never been properly studied and confirmed in bed bugs. There were several studies that reported some behavioural observations in bed bugs in the presence of insecticides. One recent study showed that insecticide-susceptible and insecticide-resistant *C. lectularius* may either avoid resting on deltamethrin-treated filter paper, or increased their movement upon direct contact with sublethal doses of deltamethrin [89]. This possibly suggested excito-repellency, a form of behavioral resistance. In another study however, Moore & Miller [48] found that insecticide-susceptible and insecticide-resistant strains of *C. lectularius* did not avoid surfaces treated with lambda-cyhalothrin. *Cimex lectularius* also showed no avoidance behavior when exposed to chlorfenapyr [48, 65, 89]. Bed bugs are highly cryptic and often hide in dark areas and in cracks and crevices. This unique behavioral characteristic may protect them from contacting insecticide on treated surfaces, or being found during the inspection process [8, 10, 90].

### Physiological resistance

In contrast to behavioral resistance, many studies have identified physiological resistance mechanisms in bed bugs (Table 1). Mamidala et al. [91, 92] recently found that metabolic detoxification, cuticular proteins, and knockdown resistance (*kdr*) mutations were associated with insecticide resistance in *C. lectularius*. Similarly, Zhu et al. [90] reported that there were *kdr* mutations and/or differentially expressed genes including metabolic genes (P450s, esterases, ABC transporters, and cuticular protein genes associated with insecticide resistance (pyrethroid resistance) in *C. lectularius*. Overall, three major physiological resistance mechanisms have been investigated in association with bed bug resistance to insecticides, namely penetration resistance, metabolic resistance, and target site insensitivity.

### Penetration resistance

Contact insecticides must pass through the insect cuticle before reaching the target site [5]. Therefore, the cuticle serves as the first line of defence against insecticides [90, 93]. Resistant insects develop cuticular barriers by evolving a thicker cuticle or by altering the cuticular structure to reduce the penetration rate of insecticides into the insect body [94, 95]. Penetration resistance may provide protection to the insects against different classes of insecticides [96, 97]. Penetration resistance also works in combination with other resistance mechanism(s), as it has been hypothesized that decreased cuticular penetration could help to ‘buy more time’ for detoxifying enzymes to metabolize the insecticide or to allow the insect to excrete the insecticide before it reaches its target [90, 98]. Penetration resistance has been reported in various insect pests, including the house fly *Musca domestica* (L.) [98–100], the German cockroach *B. germanica* [2, 101], the dengue mosquito *Aedes aegypti* (L.) [102], and the common bed bug, *C. lectularius* [94, 95, 103].

Penetration resistance plays a crucial role in insecticide resistance in bed bugs. Koganemaru et al. [94] demonstrated that the resistance ratio in the resistant Richmond *C. lectularius* strain that was topically applied with deltamethrin or beta-cyfluthrin onto the cuticle was 10^5^ greater than that when applied subcuticularly, in comparison to the insecticide-susceptible Harlan strain. However, compared with other physiological resistance mechanisms, penetration resistance remains the least understood for many insect pests. Nevertheless recently, various RNA and genomic sequencing efforts have been made to identify the putative genes associated with cuticular penetration resistance in bed bugs [90, 92–94, 104]. Bai et al. [104] analysed using the 454-pyrosequencing technique the transcriptomic sequences of *C. lectularius* from the susceptible Harlan strain and one field-collected resistant strain and identified 45 putative cuticular protein genes that were possibly associated with insecticide resistance in bed bugs. Mamidala et al. [92] detected 46 cuticular protein genes that were upregulated in the deltamethrin-resistant strains of *C. lectularius*. Five cuticular protein genes [larval cuticle protein (LCP), pupal cuticle protein (PCP), chitin synthase (CHS), chitin deacetylase (CDA), and cuticular protein analogous to peritrophin (CPAP)] were further confirmed by quantitative real-time PCR (qRT-PCR) to be possibly associated with insecticide resistance in *C. lectularius*; these genes displayed higher transcript levels in resistant strains compared to those in susceptible strains [92]. Another 19 cuticular protein genes were similarly reported to be associated with insecticide resistance in *C. lectularius*, especially pyrethroid resistance [90, 94]. Overexpression of these genes were inferred to thicken or remodel the bed bug cuticle to reduce the insecticide penetration rate [94], which could prevent or slow the insecticide from reaching the target sites on nerve cells (Fig. 1b). These results suggested that penetration resistance significantly contributes to bed bug resistance to insecticides. Unfortunately, no study on penetration resistance in *C. hemipterus* has been reported so far.

Molecular assays such as dsRNA-mediated interference (RNAi) technique may not be able to verify the association between over-expression of culticular genes and penetration resistance since the cuticle has been thickened or remodelled [90]. Nevertheless, more recently, a study examining the relationship between cuticular thickness of *C. lectularius* and insecticide residual bioassays, revealed a positive correlation between the thickened cuticle and insecticide resistance level [95]. The authors found that highly pyrethroid-resistant individuals of the Parramatta strain of *C. lectularius* possessed a significantly thicker cuticle compared with that of an insecticide susceptible strain. Also, the cuticle thickness of this resistant strain was positively correlated to time-to-knockdown in insecticide bioassays [95]. Future studies should be performed to provide direct evidence on penetration resistance in bed bugs through in vivo assay using radio-labelled insecticide [105].

### Metabolic resistance

Metabolic resistance is considered a key resistance mechanism and has been well reviewed in past literature [4–7]. Mamidala et al. [91] also provided a review of metabolic resistance in bed bugs. Based on these reports, three major groups of enzymes, namely P450s, esterases, and GSTs, as well as ABC transporters [90], are involved (Table 5) and may have a broad spectrum of activity against different insecticide classes [97]. Unfortunately, most of metabolic resistance research has been undertaken in *C. lectularius*; the studies on metabolic resistance in *C. hemipterus* are limited (Table 5).

### P450s

P450s, the most important subset of the monooxygenase system, constitute one of the largest superfamilies of proteins found in all living organisms [106–108] and plays a significant role in the detoxification of insecticides [100, 109, 110]. Recently, more than 1700 genes of P450s were characterized from various insects [7, 111, 112], and 386 contigs of P450s associated with insecticide resistance were found in hematophagus insects [91].

There are two possible mechanisms attributed to up-regulation of P450 genes [112]: (i) constitutive transcriptional overexpression (mRNA levels), in which the gene is continually transcribed, and (ii) induced transcriptional overexpression, in which the expression of the gene is induced as needed. However, the factors regulating the overexpression of P450s in these two mechanisms are less known. The first mechanism is common in many insect pests, including bed bugs. Currently, the link between insecticide resistance and constitutive overexpression of P450 genes has been shown in *C. lectularius* [90, 92, 113]. Adelman et al. [113] found that the resistance ratios to deltamethrin and β-cyfluthrin in *C. lectularius* (Richmond strain, compared with susceptible Harlan strain) were 5167 and 111, respectively. Biochemical assays revealed that the P450 activities of the Richmond strain were significantly enhanced by 41% compared to that of the Harlan strain. In addition, genetic studies revealed that three P450 genes, namely *CYP397A1* (>36-fold), *CYP6DM2* (>29-fold) and *CYP400A1* (>18-fold), were all significantly overexpressed in the Richmond strain [113]. The Richmond strain showed high resistance to pyrethroids due to overexpressed P450 genes (and possible other mechanisms as well). In addition, four P450 genes (*CYP9* [104], *CYP397A1V2*, *CYP6A2* and *CYP6A13* [92]) were found to be putatively responsible for *C. lectularius* resistance to pyrethroids. The dsRNA-mediated interference (RNAi) technique confirmed that another four P450 genes (*CYP397A1*, *CYP398A1*, *CYP6DN1* and *CYP4CM1*) were involved in *C. lectularius* pyrethroid resistance, as these genes were up-regulated in resistant strain(s) [90]. Molecular docking studies revealed that the P450 genes may confer cross-resistance to the major classes of insecticides (e.g. OCs, pyrethroids and neonicotinoids) used to control bed bugs [92]. All these findings suggest that P450-mediated detoxification plays a key role in metabolic resistance to insecticides, especially the pyrethroids.

In the second mechanism, the expression of some P450 genes can be induced by exogenous and endogenous compounds which include insecticides [110] which led to increased resistance to insecticides [114, 115]. This phenomenon had been reported in several insect pests, including *M. domestica* [116, 117] and the mosquito *Culex quinquefasciatus* (Say) [118]. Additionally, both mechanisms (constitutive and induced overexpression) can be exhibited in same insect population within an area, such as *M. domestica* [117]. However, in comparison with the constitutive overexpression, the induction associated with insecticide resistance is less well known and not described in bed bugs to date. Further studies should be conducted to better understand the mechanism of induction in bed bugs.

Currently, the mixture insecticide products which contain a neonicotinoid and a pyrethroid, are used to control bed bugs, particularly in the USA and other parts of the world [119]. Constitutive or both constitutive and induced overpression of P450 genes have been associated with neonicotinoid resistance in other insects [117, 120]. In the wake of neonicotinoid resistance in *C. lectularius* [64], further studies are warranted to determine the role of P450s, as well as potential cross-resistance in both species of bed bugs.

The reaction of the P450 system requires an electron transferred from nicotinamide adenine dinucleotide phosphate (NADPH) to the P450 heme centre by a cytochrome P450 partner enzyme, NADPH-cytochrome P450 reductase (CPR), and/or cytochrome *b*_5_ reductase in microsomal systems, and by an adrenodoxin-like ferredoxin coupled to an adrenodoxin reductase in mitochondrial systems [121–124]. Recently, Zhu et al. [122] sequenced and characterized the gene of CPR from *C. lectularius* (*ClCPR*) and found that RNAi suppressed the expression of ClCPR, which led to the resistant CIN-1 strain (a field-collected *C. lectularius* strain collected in 2005 in Cincinnati, OH) showing increased susceptibility to deltamethrin. This finding verified at least a partial role of CPR in P450-mediated detoxification and indicated that P450-mediated metabolic resistance to pyrethroids occurred in the CIN-1 strain.

Piperonyl butoxide (PBO), a primary inhibitor of some cytochrome P450 monooxygenases, is used to characterize the possible involvement of P450-mediated detoxification as a resistance mechanism [125–127]. PBO could be included in formulations as a synergist of pyrethroid-based insecticides [128, 129] to enhance their efficacy. Romero et al. [128] used PBO to determine the role of P450s in deltamethrin resistance in two highly resistant *C. lectularius* strains (CIN-1: Cincinnati, OH, USA; WOR-1, collected in 2007 in Worcester, MA, USA). The results showed that the resistance level of CIN-1 and WOR-1 reduced from >2588-fold to 174-fold and from >2588-fold to 39-fold after treatment with PBO, respectively, when compared to the corresponding results obtained with the Fort Dix (= Harlan) susceptible strain [128]. These results indicated that P450s contribute in part to the deltamethrin resistance in these strains of *C. lectularius*. Similar synergism studies were performed by How & Lee [129] and Lilly et al. [127] for two Southeast Asian *C. hemipterus* strains and four Australian *C. lectularius* strains, respectively. Both studies supported that P450-mediated metabolic resistance to pyrethroids occurred in bed bugs.

### Esterases

Esterases confer resistance to carbamates and OPs in many insect species [2, 4, 5, 7, 130–132] as well as to pyrethroids [127, 133], mainly due to the activity of carboxylesterases [134, 135] and only in a few rare cases by arylesterases (aromatic esterases) [2].

Esterases (especially carboxylesterases) mediate metabolic resistance by two main mechanisms: (i) increased level of gene expression (quantitative change), and (ii) mutations in coding gene sequences (qualitative changes) [4, 7]. In the first mechanism (quantitative change), resistant insects overproduce non-specific esterases or carboxylesterases by gene up-regulation to quickly sequester the insecticides (e.g. carbamates and OPs) [133]. This mechanism has been documented in numerous insect species including mosquitoes, cattle ticks, aphids, cockroaches [4], and both *C. hemipterus* [56] and *C. lectularius* [64, 90, 113]. Adelman et al. [113] recently reported a significant increase in the general esterase activity (at least by 35%) based on the biochemical assays in a highly resistant Richmond *C. lectularius* strain, when compared to that of the susceptible Harlan strain. Their subsequent findings based on genetic studies (via RNA sequencing and relative gene expression based on qRT-PCR) identified that two esterase-encoding genes, *CE3959* and *CE21331*, were significantly overexpressed in the Richmond strain. These findings suggest that *CE3959* and *CE21331* may be candidate genes contributing to esterase-mediated resistance in *C. lectularius*. Zhu et al. [90] also found that the gene *CLCE21331* (also known as *CE21331*) was associated with *C. lectularius* pyrethroid resistance, due to overexpression in resistant strains. They subsequently determined that the gene *CLCE21331* showed maximum up-regulation (>50-fold) in most field populations (76.2% of 21 populations) compared with the susceptible LA-1 *C. lectularius* strain (collected in 2006 in Los Angeles, CA, USA [30]), that strongly suggests the importance of esterase-mediated metabolic resistance in bed bugs. Karunaratne et al. [56] found that elevated esterase mechanisms were present in *C. hemipterus* populations based on biochemical assays, and elevated levels of general esterases were similarly found to be associated with resistance to the neonicotinoids in *C. lectularius* [64]. However, further studies including the use of metabolism studies [105], should be attempted to experimentally validate the specific gene(s) encoding esterase mediated mechanism in bed bugs. Besides the biochemical assays and genetic studies on esterase-mediated resistance, researchers also have used bioassays in combination with synergists to investigate the activity levels of general esterases in resistant bed bug populations. Hardstone et al. [136] selected PBH (3-phenoxybenzyl hexanoate, a surrogate substrate for carboxylesterases and oxidases) as a metabolic synergist to suppress resistance to pyrethroids in *C. lectularius*. The authors found that PBH synergized the action of deltamethrin 6-fold on an insecticide-susceptible *C. lectularius* strain (FL-BB, collected from Gainesville, FL, USA, more than 20 years ago), and was 2.8-fold more synergistic than PBO. These findings suggested that esterases were involved in *C. lectularius* metabolic detoxification of deltamethrin. Similarly, Lilly et al. [127] employed a novel synergist, EN16/5–1 (6-[2-(2-butoxyethoxy) ethoxymethyl]-5-propyl-2, 3-dihydrobenzofuranby), which mainly inhibits the activity of esterases, to determine if esterase-mediated pyrethroid resistance exists in *C. lectularius*. They found that the resistance in three of four *C. lectularius* strains to deltamethrin was significantly suppressed by EN16/5–1, which strongly suggested that esterases conferred metabolic resistance to *C. lectularius* in Australia.

In the second mechanism (qualitative changes), resistant insects can increase esterase-mediated metabolism due to a single point mutation or substitution in the structural genes. For example, the *LcaE7* gene of the sheep blowfly *Lucilia cuprina* (Wiedemann) encodes a carboxylesterase. A single point mutation on the *LcaE7* gene changes glycine at residue site 137 to an aspartic acid and then converts the carboxylesterase to an organophosphorus hydrolase that confers organophosphorus resistance [137, 138]. However, limited reports about this mechanism are available worldwide [7]. This mechanism still is a candidate mechanism and yet to be reported in bed bugs.

### GSTs (candidate resistance mechanism)

GSTs mediate metabolic resistance to organophosphates, chlorinated hydrocarbons, and pyrethroids through catalyzing the conjugation of electrophilic compounds by reduced glutathione (GSH) [7, 139–141]. On the other hand, some insect GSTs catalyse a dehydrochlorination reaction by using GSH as a cofactor rather than as a conjugate [7, 141, 142]. GSTs are also involved in detoxification via xenobiotic binding, intracellular transport of endogenous lipophilic compounds, or sequestration [7].

Using biochemical assays, Karunaratne et al. [56] found that DDT resistance in a Sri Lankan *C. hemipterus* strain was associated with high GST levels, as dehydrochlorination of DDT by GSTs is a major route of detoxification in insects [141]. Adelman et al. [113] found that one GST gene (*gsts1*) was putatively associated with pyrethroid resistance in the Richmond *C. lectularius* strain due to up-regulated transcription, and three other GST genes were similarly identified by Mamidala et al. [92]. However, further studies on transgenic expression (such as those in *Drosophila* flies [143]) and metabolism studies [105], are urgently warranted to empirically confirm this mechanism in bed bugs.

### ABC transporters

ATP-binding cassette transporters (ABC transporters) are one of the largest classes of transporters that are responsible for the ATP-powered translocation of many substrates across membranes. The function of ABC transporters is as either importers, which bring nutrients and other molecules into cells, or as exporters, which pump toxins, drugs and lipids across membranes [144–146]. In addition, ABC transporters were found to increase the efficiency of toxin removal from the targeted site. Hence, ABC transporters have been associated with resistance to major insecticide classes (Table 5) although not directly related to the detoxification of the compounds. In bed bugs, Mamidala et al. [92] suspected that ABC transporters were involved in metabolic resistance in *C. lectularius* due to overexpression of the genes encoding ABC transporters. Zhu et al. [90] confirmed the role of ABC transporters (e.g. *Abc 8* and *Abc 9*) mediated metabolic resistance to pyrethroids (β-cyfluthrin) in *C. lectularius*, via RNAi. In addition, the authors also identified this mechanism was widespread in the field populations (20/21) [90].

Biochemical and molecular assays have identified that P450s, esterases, and GSTs are associated with bed bug insecticide resisance. Synergism studies [95] and molecular assays based on RNAi [90] have confirmed that P450s and esterases as well as ABC transporters mediated resistance mechanisms exist to pyrethroids in *C. lectularius*. However, most of these mechanisms have yet to be demonstrated in *C. hemipterus.* In addition, further studies specifically involving metabolism experiments to empirically demonstrate the disappearance of the parent compound and an increase of metabolites in resistant strains would be helpful in clarifying the role of these mechanisms in bed bugs.

### Target site insensitivity

Insecticides such as OPs, carbamates, DDT, and pyrethroids affect specific target sites (e.g. AChEs, VGSC, GABA receptor) (Table 5) that typically are associated with the insect nervous system [147]. Insecticide-resistant insects have evolved modifications at these target sites that allows for normal neurological function to occur, despite the presence of the toxicant. The four main types of target site insensitivity mechanisms in resistant insects are: (i) knockdown resistance (*kdr*-type), which causes resistance to pyrethroids and DDT; (ii) altered AChEs, which confer resistance to OPs and carbamates; (iii) insensitive GABA receptors (also known as *rdl*-mutation), which provide resistance to cyclodienes and phenylpyrazoles; and (iv) altered nAChRs, which confer resistance to neonicotinoids [6, 147, 148].

### *Kdr*

VGSCs are essential for normal transmission of nerve impulses [149]. DDT and pyrethroids act on or bind to the VGSC proteins to disrupt the process, which is followed by knockdown, paralysis, and eventually death of the insect. Many insect pests have evolved moderate to high levels of resistance to DDT and pyrethroids by reducing target site sensitivity (so-called *kdr*) [150]. The first case of reduced neuronal sensitivity to DDT was reported in the 1950s in *M. domestica* [151]. The *kdr*-resistance is a recessive trait that confers cross-resistance to most pyrethroids as well as DDT and its analogues [46, 152].

**Fig. 2:**
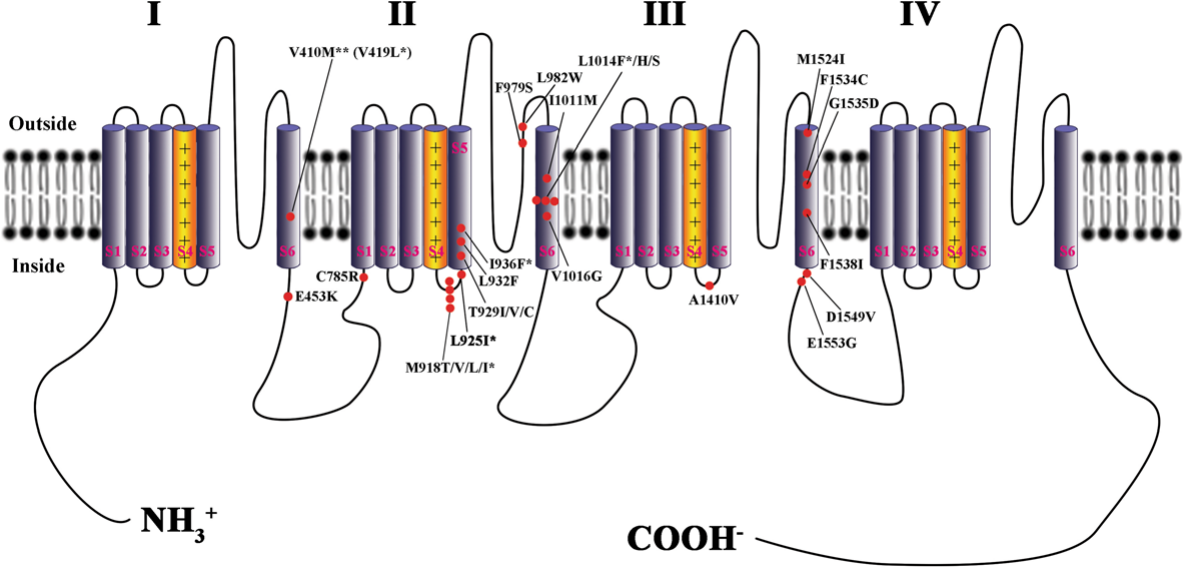
*kdr* mutations in insect voltage-gated sodium channels implicated in pyrethroid/DDT resistance. All information of *kdr* mutation sites came from Davies & Williamson [150], Zhu et al. [157] and Dang et al. [17, 54]. I, II, III and IV, four homologous repeat transmembrane domains. One asterisk indicates *kdr* mutations (or putative *kdr* mutations) identified from both the common bed bug *C. lectularius* (V419L, valine to leucine; L925I, leucine to isoleucine; putative I936F, isoleucine to phenylalanime) and the tropical bed bug *C. hemipterus* (putative M918I, methionine to isoleucine; putative L1014F, leucine to phenylalanime). Two asterisks: V410 found in the tobacco budworm *Heliothis virescens* (F.) and V419 found in *C. lectularius* are the same residue

The VGSC gene from *D. melanogaster* was originally cloned and sequenced in the late 1980s [153]. This study revealed how to sequence the VGSC gene of both resistant and susceptible insects. Several studies showed that *kdr*-type resistance to DDT and pyrethroids results from a single or multiple point mutations (also known as *kdr* mutations) in coding sequences of VGSC in various insect pests (Fig. 2) [46, 150, 154, 155], including bed bugs [17, 54, 156, 157]. Yoon et al. [156] first cloned and sequenced the coding gene of *C. lectularius* VGSC from both insecticide resistant and susceptible strains in the USA. Two *kdr* mutations (V419L: valine 419 to leucine, and L925I: leucine 925 to isoleucine) were linked to confer pyrethroid resistance in *C. lectularius* [156]*.* These two *kdr* mutations (either one or both) were found to be widely distributed across the USA in *C. lectularius* (88% of 117 strains [157] and 85.7% of 21 strains [90] in different studies). One hundred percent of the tested *C. lectularius* collected from Paris, France [158] and Berlin, Germany [159] only contained L925I. Of *C. lectularius* collected from various locations in Australia (25 strains), 96% possessed L925I [55], while from Israel (12 strains), 100% had L925I, of which, a few specimens from both countries had additional V419L [54, 160]. Based on the various genotypes of V419L and L925I mutations of 110 field-collected strains of *C. lectularius* in the USA, Zhu et al. [157] identified four haplotypes; haplotype A (without V419L and L925I mutations; 15.5% [17/110]), haplotype B (only L925I; 40.9% [45/110]), haplotype C (V419L and L925I; 40.9% [45/110]) and haplotype D (V419L; 2.7% [3/110]).

Recently, a novel mutation, I936F (isoleucine 936 to phenylalanine), was identified in the VGSC gene of one field-collected *C. lectularius* strain (from Adelaide in Australia) that was linked to low levels of resistance to d-allethrin [54]. This novel mutation was also found in museum preserved *C. lectularius* specimens collected over 1994–2002 from four disparate locations (4/7) and one field-collected Perth *C. lectularius* strain (collected in 2007), all from Australia [54]. Interestingly, the museum preserved specimens and the Adelaide strain did not have the *kdr* V419L and L925I mutations (haplotype A). Only the Perth strain had the L925I mutation (haplotype B). The novel I936F mutation was probably once relatively prevalent in preserved Australian *C. lectularius* specimens (without V419 L and L925I) but largely disappeared after 2004, when the knowledge of bed bug control practices was improved [15, 54]. It is possible that I936F only conferred low levels of resistance and the populations with the mutation did not survive, as the Adelaide strain showed relatively higher susceptibility to d-allethrin than that in other strains with L925I or both V419L and L925I [54]. Although I936F mutation confers low levels of resistance, it may help other mutation(s) or other resistance mechanism(s) to evolve. The findings that the Australian Perth *C. lectularius* strain had L925I and both L925I and I936F mutations [54] may support this scenario.

Another recent study has identified four novel mutations in the VGSC genes of *C. hemipterus* collected from multiple countries, including Australia [L899V (leucine 899 to valine), M918I (methionine 918 to isoleucine), D953G (aspartic acid 953 to glycine) and L1014F (leucine 1014 to phenylalanine)], Thailand (M918I, D953G and L1014F), India (M918I and L1014F), Malaysia (L1014F) and Kenya (L1014F), of which, two mutations (M918I and L1014F) were associated with high resistance to pyrethroids in *C. hemipterus* [17]; however the presence of other resistance mechanisms were not excluded. These two sites are known to confer pyrethroid resistance in a wide range of insect pests (Fig. 2) [150, 157]. The I936F, M918I and L1014F mutations could be verified as *kdr* mutations in bed bugs by functional expression of each mutation individually and in combination using the *Xenopus* oocyte expression system with two-electrode voltage-clamp electrophysiology [161], although this has yet to be undertaken.

The reports of the various mutations in the VGSC genes suggest that *kdr*-resistance in *C. lectularius* and *C. hemipterus* is widespread. Seong et al. [162] found that deltamethrin resistance levels increased in *C. lectularius* with increasing frequency of the resistance allele (e.g. L925I mutation). The M918I mutation, which was always found together with the L1014F mutation, probably plays a synergistic role in enhancing pyrethroid resistance in *C. hemipterus* [17]. These findings may suggest that multiple *kdr* mutations play a significant role in bed bug resistance to pyrethroids and DDT.

It is noteworthy that the *kdr* mutations found in the two sympatric species (*C. hemipterus* and *C. lectularius*) have occurred at different regions in the genome and not identical between the two species. In contrast to *C. lectularius*, *kdr* mutations in *C. hemipterus* may likely have occurred from the malaria control programs (e.g. the widespread use of pyrethroid-treated bed-nets and indoor residual wall sprays of DDT/pyrethroid) in the tropics [12, 28, 55, 56]. *Cimex hemipterus* from Kenya (with L1014F), Malaysia (L1014F), India (M918I and L1014F) had similar mutations, in which, these mutations were also found in malaria vectors of *Anopheles* spp. [46, 163]. The presence of *kdr* mutations in *Anopheles* spp. could have severe consequences for the sustainable use of pyrethroids, especially when pyrethroids are presently used for treating bed nets [164]. On the other hand, mutations may be completely random, and the different *kdr* mutations occurring between *C. lectularius* and *C. hemipterus* may be a function of this randomness and completely unrelated to the use of a particular insecticide.

### Altered AChEs (candidate resistance mechanism)

The first report of insensitivity of AChE to OPs and carbamates was reported in the spider mite *Tetranychus urticae* (Koch), and subsequently reduced sensitivity of AChE was reported as a common OP/carbamate resistance mechanism in many insect species [4, 165–168].

The genetic study of the gene sequence and genomic structure encoding for AChE (symbol: *Ace*) in different insects revealed that sensitivity of AChE to inhibition was reduced by altered amino acids caused by point mutations on the *Ace* gene [169, 170], which is referred to as ‘altered AChE’. For example, the malathion-resistant MH19 strain of *D. melanogaster* was found to contain a single amino acid substitution, F368Y (phenylalanine 368 to tyrosine), caused by a point mutation in the *Ace* gene. This point mutation was found to confer resistance to malathion by P-element transformation experiments [165]. Subsequently, three other point mutations [phenylalanine 115 to serine (F115S), isoleucine 199 to threonine/valine (I199T/V), and glycine 303 to alanine (G303A)] in the *Ace* gene sequence of *D. melanogaster* were identified [171]. These four mutations in *D. melanogaster* associated with resistance to OPs and carbamates were verified using the *Xenopus laevis* oocyte expression system [171]. Furthermore, other mutations in the gene encoding AChE have been found in other insects [4, 170] which reduce the degree of AChE inhibition by insecticides. Additionally, like the *kdr*-type resistance, insecticide resistance due to altered AChE may produce a broad range of cross-resistance among OPs and carbamates [172, 173].

Several studies had identified resistance to OPs and carbamates in both *C. lectularius* [11, 44, 47, 53] and *C. hemipterus* [12, 56, 129]. Karunaratne et al. [56] reported that 29–44% of Sri Lankan *C. hemipterus* populations showed target site AChE insensitivity, and this may be responsible for resistance to both OPs and carbamates.

Recently, Seong et al. [168] identified and characterized the full-length-cDNA sequences encoding two AChEs (*CAChE1* and *CAChE2*) from *C. lectularius*. Hwang et al. [174] examined the molecular and enzymatic properties of these two AChEs. The relatively higher correlation between in vitro *ClAChE1* inhibition and in vivo toxicity suggested that *ClAChE1* is the more relevant toxicological target for OPs and carbamates in *C. lectularius*. These findings offer valuable insights into altered AChE-mediated resistance in bed bugs, although most of these AChE-inhibitor insecticides have been prohibited for usage indoor against bed bugs in many parts of the world.

### Insensitive GABA receptor (candidate resistance mechanism)

The GABA-gated chlorine channel, which is also known as the GABA receptor, is the validated target for cyclodiene (e.g. dieldrin) and phenylpyrazole (e.g. fipronil) [175–178]. Resistance to cyclodienes in several insect pests is caused by a single mutation [A302S/G: alanine 302 to serine/glycine (residue 302 in *D. melanogaster*, and residue 296 in *An. gambiae* are the same residue)] in the GABA receptor [147, 179, 180]. The replacement of alanine 302 affects the binding site and destabilizes the preferred conformation of the receptor [181]. Furthermore, an additional mutation (V327I: valine to isoleucine) was detected in the same gene, and it was consistently associated with the mutation A296S (alanine 296 to serine) in resistant anophelines [182].

Several studies had identified resistance to dieldrin in both *C. lectularius* and *C. hemipterus* [38], but both cyclodienes and phenylpyrazoles are currently not legally allowed to be used against bed bugs in most countries. Recently, the genome sequencing of *C. lectularius* was completed, and the resistance to dieldrin (*Rdl*) gene encoding the GABA receptor was identified [93]. This cyclodiene target site is a target site for phenylpyrazoles (e.g. fipronil) as well. Therefore, these data may provide an important clue to reveal the resistance mechanism of insensitive GABA receptor to both cyclodienes and phenylpyrazoles in bed bugs in the future.

### Altered nAChRs (candidate resistance mechanism)

Neonicotinoids are widely used to control a broad range of sucking insect pests in plants [58, 120]. Neonicotinoids act selectively on the insect central nervous system as agonists of the nAChRs, opening the channel and causing continuous depolarisation and firing of postsynaptic neurons resulting in paralysis and death [183]. With the increase of neonicotinoid usage, resistance to these insecticides has increasingly been reported, not only due to metabolic detoxification mechanism, such as P450s, but also due to the target-site mutation(s) on nAChRs [120, 184]. For example, the mutation R81T (arginine 81 to threonine) present in the β1 nAChR subunit confers high levels of resistance to imidacloprid in cotton-melon aphid, *Aphis gossypii* (Glover) [185] and green peach aphid, *Myzus persicae* (Sulzer) [186]. More recently, Romero & Anderson [64] reported high levels of neonicotinoid resistance in *C. lectularius*. Although metabolic resistance including general esterases may be involved [64], the role of altered nAChR has never been confirmed in the bed bug.

### Symbiont-mediated insecticide resistance (candidate resistance mechanism)

Symbiotic relationships between animals and microorganisms are common in nature. Various microorganisms have established associations with animal hosts through parasitism, mutualism and commensalism, or by ectosymbiosis and endosymbiosis [187]. In insects, bacterial symbionts, such as *Wolbachia*, commonly associated with hosts, can manipulate insect host reproduction and nutritional mutualism [188]. Interestingly, bacterial symbionts have been identified to be involved in insecticide resistance in some insect pests, which is termed ‘symbiont-mediated’ insecticide resistance [189, 190]. Kikuchi et al. [189] found that fenitrothion-degrading *Burkholderia* endosymbiont strains established a specific and beneficial symbiosis with the soybean bug *Riptortus pedestris* (F.) and conferred fenitrothion resistance. Apart from *Burkholderia*, a variety of organophosphorus degraders have been isolated and characterized from many bacterial genera [191]. In addition, bacterial symbionts can be involved in insect resistance to biological agent such as *Bacillus thuringiensis* Berliner [192] and the parasitoid wasp *Aphidius ervi* Haliday [193]. A recent study suggested a correlation between the high bacterial densities (e.g. *Arsenophonus*, *Rickettsia*, and *Wolbachia*) in whitefly *Bemisia tabaci* (Gennadius) and the insect’s ability to detoxify toxic compounds such as insecticides (e.g. thiamethoxam, imidacloprid, pyriproxyfen and spiromesifen) [194].

Recently, molecular studies identified various symbionts, especially the bacterial symbionts, in bed bugs [104, 188, 195–197], although the first endosymbiont, *Rickettsia lectularia* Arkwright et al. (= *Symbiotes lectularius*), was documented by light microscopy in *C. lectularius* almost 90 years ago [198]. Hypsa & Aksoy [195] first isolated two symbionts, *Wolbachia* (an alpha-proteobacterium) and a BEV-like symbiont (an unnamed gamma-proteobacterium) from ovary tissue of *C. lectularius*, which were later found to be responsible for manipulating reproduction of bed bugs [196]. Subsequently, *Wolbachia* was found to play an essential role in nutritional mutualism in *C. lectularius* [188]. A transcriptomic study by Bai et al. [104] identified a total of 14.24% of non-insect transcriptomic sequences (e.g. non-insect eukaryotes, 11.16%, fungi, 1.78%, bacteria, 1.21%, viruses, 0.04%, Archaea, 0.02%, and artificial sequences, 0.03%) in *C. lectularius* that probably belongs to various symbionts or pathogens in bed bugs. In fact, a genomic study by Benoit et al. [197] further demonstrated various sequences from multiple bacterial symbionts and/or possible pathogens, or opportunists in *C. lectularius*, for instance, *Arsenophous*, *Wolbachia*, *Sodalis*, *Hamiltonella*, *Peptoclostridium*, *Methanococcus*, *Serratia*, *Shewanella*, and 81 other genera. Despite the presence of multiple functional symbionts in bed bugs, their potential role in mediating insecticide resistance in bed bugs is still undocumented and should be considered in future studies.

## Monitoring insecticide resistance

Over the last few years, there have been many reports of insecticide resistance in bed bugs worldwide. Monitoring insecticide resistance status and resistance mechanisms in bed bugs is a proactive and essential approach to determine proper insecticide usage and to provide early warning for the need to modify chemical control strategies. Numerous studies on the methods of detecting and documenting insecticide resistance in pest populations have been evaluated. The three major methods of monitoring insecticide resistance are (i) conventional toxicity bioassays, (ii) biochemical assays, and (iii) molecular assays (Table 6).

**Table 6:** Methods used to monitor for insecticide resistance in bed bugs (*Cimex* spp.)

Method^a^	Advantages	Disadvantages
Bioassays via dose/concentration-response assays	Provide levels of resistance	Require a susceptible strain, need large numbers of live bed bugs; regardless of mechanism(s)
Bioassays via discriminating concentration/dose assays	Standardized (e. g. WHO susceptibility test kits); simple to perform	Provide no information about levels of resistance and type of resistance mechanism(s) (except when using synergists); lack sensitivity
Biochemical assays	Require a small sample size; sensitive; provide indirect evidence on specific resistance mechanism(s)	Require sophisticated and costly equipment and the technology which pest managers do not have ready access to; require materials to be kept frozen
Molecular assays	Require a small sample size; very sensitive; provide informations on specific resistance mechanism(s); can detect resistance alleles (e. g. *kdr* mutations), even from dead body; could develop specific molecular markers to detect specific mechanism(s)	Require specific and costly equipment, high ongoing costs and the technology which pest managers may not have access to; costly reagents, require materials to be kept frozen

^a^Information from Ranson et al. [163], Seong et al. [162] and Dang et al. [16]

### Conventional toxicity bioassays

The standard bioassay that is routinely used to detect insecticide resistance involves collecting insects from the field and rearing them until sufficient numbers are available for testing. Mortality of nymphs or adults is then assessed after exposure to a range of doses of an insecticide. Subsequently, the LD_50_, LC_50_ or LT_50_ values are determined using probit analysis. The results from field populations are then compared with those from a susceptible population, and a resistance ratio is calculated to estimate the susceptibility of field populations. The susceptibility of recent collected bed bugs to major insecticide classes (such as pyrethroids, OPs, carbamates, and neonicotinoids) have been evaluated using bioassays (Table 7) [30, 44, 48, 53, 64].

**Table 7:** Published discriminating doses and susceptibility baselines used to detect insecticide resistance in bed bugs (*Cimex* spp.)

Insecticide	Discriminating concentration	Assessment	Susceptibility baseline		Reference
			LC_50_/LD_50_	LC_90(99)_/LD_90(99)_	
*C. lectularius*					
DDT^a^	4%	5 d^f^	–	–	[39]
Dieldrin^a^	0.8%	2 d^f^	–	–	[39]
Fenitrothion^b^	1%	5 h^f^	–	–	[39]
Malathion^b^	5%	16 h^f^	–	–	[39]
Trichlorfon^b^	1%	5 h^f^	–	–	[39]
Propoxur^c^	0.8%	24 h^f^	–	–	[39]
Permethrin^d^	0.25%	C^f^	–	–	[39]
Deltamethrin^d^	0.025%	C^f^	–	–	[39]
Dichlorvos^b^	–	24 h^f^	2.9 ppm	5.7 ppm	[202]
Pirimiphos-methyl^b^	–	24 h^f^	13.5 ppm	29.8 ppm	[202]
λ-cyhalothrin^d^	–	24 h^f^	22.2 ppm	357.7 ppm	[202]
Permethrin^d^	–	24 h^f^	71.4 ppm	201.7 ppm	[202]
Bendiocarb^c^	–	24 h^f^	47.1 ppm	95.9 ppm	[202]
Malathion^b^	–	24 h^f^	92 ppm	245 ppm	[202]
Carbaryl^c^	–	24 h^f^	166.3 ppm	245 ppm	[202]
Tetrachlorvinphos^b^	–	24 h^f^	252 ppm	472.7 ppm	[202]
Bendiocarb^c^	35.3 M	48 h^f^	–	35.3 M	[47]
α-cypermethrin^d^	23.1 M	48 h^f^	–	23.1 M	[47]
Deltamethrin^d^	0.06%	C^g^	–	–	[48]
Deltamethrin^d^	1300 M (30 × LC_99_)	24 h^f^	3.1 M	44 M	[30]
λ-cyhalothrin^d^	–	24 h^f^	2.16 M	66 M	[30]
Deltamethrin^d^	1%	C^f^	–	–	[156]
Deltamethrin^d^	–	24 h^f^	1.4 M	19.2 M	[201]
Bendiocarb^c^	–	24 h^f^	6.5 M	38.1 M	[201]
Pirimphos-methyl^b^	–	T, 24 h	0.11 U	–	[49, 52]
Imidacloprid^e^	–	T, 24 h	0.0057 U	–	[49, 52]
Bendiocarb^c^	–	T, 24 h	0.027 U	–	[49, 52]
Permethrin^d^	–	T, 24 h	0.00044 U	–	[49, 52]
Deltamethrin^d^	–	T, 24 h	0.00057 U	–	[49, 52]
Deltamethrin^d^	–	1 h^f^	18.1 ppm	81.8 ppm	[162]
Deltamethrin^d^		12 h^f^	3.2 ppm	26 ppm	[162]
λ-cyhalothrin^d^		1 h^f^	17.7 ppm	87 ppm	[162]
λ-cyhalothrin^d^		12 h^f^	3.4 ppm	30 ppm	[162]
Deltamethrin^d^	1300 M (30 × LC_99_)	24 h^f^	–	–	[157]
Deltamethrin^d^	–	IT, 24 h	0.00003 U	–	[113]
β-cyfluthrin^d^	–	IT, 24 h	0.00004 U	–	[113]
DDT^a^	4%	C ^f^	–	–	[12]
Dieldrin^a^	0.8%	C ^f^	–	–	[12]
Bendiocarb^c^	0.1%	C ^f^	–	–	[12]
Propoxur^c^	0.1%	C ^f^	–	–	[12]
Malathion^b^	5%	C ^f^	–	–	[12]
Fenitrothion^b^	1%	C ^f^	–	–	[12]
Cyfluthrin^d^	0.15%	C ^f^	–	–	[12]
Deltamethrin^d^	0.05%	C ^f^	–	–	[12]
Permethrin^d^	0.75%	C ^f^	–	–	[12]
λ-cyhalothrin^d^	0.05%	C ^f^	–	–	[12]
Etofenprox^d^	0.5%	C ^f^	–	–	[12]
Permethrin^d^	2.56 U (1.6 × LC_99_)	T, 24/48 h	0.159 U	1.65 U	[53]
Permethrin^d^	57.6 M	40 min^h^	–	–	[53]
Deltamethrin^d^	19.6 M	40 min^h^	–	–	[53]
Chlorpyrifos^b^	0.2 U (2 × LC_99_)	T, 24/48 h	0.03 U	0.1 U	[53]
Chlorpyrifos^b^	53 M	40 min^h^	–	–	[53]
Malathion^b^	0.007% (2 × LC_95_)	T	–	–	[248]
Diazinon^b^	0.02% (2 × LC_95_)	T	–	–	[248]
Trichlorfon^b^	1.4% (2 × LC_95_)	T	–	–	[248]
Chlorpyrifos^b^	0.014% (2 × LC_95_)	T	–	–	[248]
Permethrin^d^	0.03% (2 × LC_95_)	T	–	–	[248]
Cypermethrin^d^	0.00008% (2 × LC_95_)	T	–	–	[248]
α-cypermethrin^d^	0.000001% (2 × LC_95_)	T	–	–	[248]
Deltametrhrin^d^	0.00008% (2 × LC_95_)	T	–	–	[248]
λ-cyhalothrin^d^	0.00005% (2 × LC_95_)	T	–	–	[248]
Imidacloprid^e^	0.0015% (2 × LC_95_)	T	–	–	[248]
Acetamiprid^e^	0.0044% (2 × LC_95_)	T	–	–	[248]
Deltamethrin^d^	1300 M (30 × LC_99_)	24 h^f^	–	–	[92]
Deltamethrin^d^	0.06 U (100 × LD_50_)	T, 24 h	0.0006 U	–	[122]
Deltamethrin^d^	–	24 h^f^	30 M	–	[90]
Deltamethrin^d^	–	T	0.0004 U	–	[94]
β-cyfuthrin^d^	–	T	0.00308 U	–	[94]
Deltamethrin^d^	–	24 h^f^	2.58 M		[159]
d-allethrin^d^	40 mg/mat	24 h^i^	–	–	[16, 54]
Imidacloprid^e^	–	T, 72 h	0.0023 U	–	[64]
Acetamiprid^e^	–	T, 72 h	0.0003 U	–	[64]
Thiamethoxam^e^	–	T, 72 h	0.0019 U	–	[64]
Dinotefuran^e^	–	T, 72 h	0.0145 U	–	[64]
*C. hemipterus*					
DDT^a^	2%	1 h^f^	–	–	[39]
α-cypermethrin^d^	20 M	72 h^j^	–	–	[55]
Permethrin^d^	200 M	72 h^j^	–	–	[55]
Permethrin^d^	0.75%	72 h^f^	–	–	[55]
DDT^a^	2%	24 h^f^	–	–	[56]
Malathion^b^	5%	16 h^f^	–	–	[56]
Propoxur^c^	0.8%	24 h^f^	–	–	[56]
Deltamethrin^d^	0.025%	C^f^	–	–	[56]
Permethrin^d^	0.25%	C^f^	–	–	[56]
DDT^a^	4%	C^f^	–	–	[12]
Dieldrin^a^	0.8%	C^f^	–	–	[12]
Bendiocarb^c^	0.1%	C^f^	–	–	[12]
Propoxur^c^	0.1%	C^f^	–	–	[12]
Malathion^b^	5%	C^f^	–	–	[12]
Fenitrothion^b^	1%	C^f^	–	–	[12]
Cyfluthrin^d^	0.15%	C^f^	–	–	[12]
Deltamethrin^d^	0.05%	C^f^	–	–	[12]
Permethrin^d^	0.75%	C^f^	–	–	[12]
λ-cyhalothrin^d^	0.05%	C^f^	–	–	[12]
Etofenprox^d^	0.5%	C^f^	–	–	[12]
d-allethrin^d^	40 mg/mat	24 h^i^	–	–	[16, 17]

*Abbreviations*: *T* topical application, *IT* injection topical application [113], *C* continuous exposure, *d* days, *h* hours, *M* mg AI m^−2^, *U* μg μL^−1^ or μg insect^−1^

^a^Chlorinated hydrocarbons

^b^OPs

^c^Carbamates

^d^Pyrethroids

^e^Neonicotinoids

^f^Surface contact on filter paper

^g^Surface contact on Hardboard panels

^h^Surface contact on glass plates

^i^Surface contact on mosquito mat

^j^Surface contact on netting

However, bioassays can be difficult to undertake. Normally this method requires a relatively large number of live bed bugs for the test, and obtaining such numbers is not always possible, especially when the number of bed bugs collected from field infestations can be relatively small [17, 162]. In addition, a standard susceptible strain is required for comparison, but many organisations do not have access to such a strain. A few susceptible *C. lectularius* strains are maintained in laboratories around the world, such as the Ft. Dix strain (= Harlan strain, established in 1973) [30, 113], FL-BB strain (early 1990s) [30, 136], LA-1 strain (2006) [30, 90], UBA strain of the Federal Environment Agency (since 1947) [159], and Monheim (Germany) strain (late 1960s) [53], but to date, no susceptible *C. hemipterus* strain is available. Other traditional bioassays, such as the use of discriminating concentrations which are based on previous studies on the dose–response curves of susceptible strains, could be an alternative option [16, 39], if there is no susceptible strain or sufficient number of live bed bugs available for testing. However, a single discriminating dose could only indicate whether resistance is present, but not the degree of resistance.

Most insecticide resistance monitoring depends on traditional bioassays, which use a fixed insecticide concentration (e.g. discriminating/diagnostic concentration) for a pre-determined exposure time in a chamber or on a filter paper impregnated with insecticides. The results are reported as percentage mortality and/or knockdown effect. For example, the diagnostic concentration (or WHO susceptibility test kit) defined by the World Health Organization (WHO) (e.g. twice the concentration/dosage that kills 100% of the susceptible insect strain) is widely used to determine the susceptibility or resistance to major classes of insecticides in mosquito vectors [38, 39]. Several studies have investigated bed bug resistance using this method (Table 7). Myamba et al. [55] adapted the WHO mosquito test kit [199] to detect pyrethroid resistance in *C. hemipterus* in Tanzania. Karunaratne et al. [56] also adapted the WHO method [39] to determine resistance to several insecticides in *C. hemipterus* in Sri Lanka. Tawatsin et al. [12] examined the insecticide resistance of both *C. lectularius* and *C. hemipterus* in Thailand using the WHO test kit [200].

Similar to the WHO methods, many studies established the baseline susceptibility data (e.g. LC_99_/LD_99_) for insecticides that results in 99% or more mortality of susceptible bed bug strain(s) (Table 7) [30, 47, 53, 201, 202]. These data can serve as guidelines for selecting discriminating concentrations to screen for bed bug resistance to insecticides, even in the absence of a susceptible strain. For example, Boase et al. [47] established two different discriminating concentrations that produced 99% mortality in three susceptible *C. lectularius* strains in the United Kingdom to detect resistance to bendiocarb (carbamates) and alpha-cypermethrin (pyrethroids), respectively. Romero et al. [30] used a discriminating concentration (10-fold greater than the labelled rate of active ingredient in the commercial product and nearly 30-fold the dose required to kill 100% of the susceptible Ft. Dix *C. lectularius* strain) to evaluate resistance to deltamethrin in third-to-fifth instar *C. lectularius* of 10 field-collected populations. Zhu et al. [157] also used this discriminating concentration to assess resistance to pyrethroids in 17 *C. lectularius* populations. Kilpinen et al. [53] determined the *C. lectularius* resistance to permethrin by the discriminating concentration of 1.6-fold LD_99_ and chlorpyrifos by the concentration of 2-fold LD_99_, respectively. However, if the discriminating concentrations used are excessively high, this could potentially mask the detection of resistance, especially when the resistance level is still relatively low. Early detection of resistance is only possible if the diagnostic concentration is low enough (or a dose response curve undertaken). Otherwise, resistance could only be discovered after widespread field control failures are reported.

Once the resistance status is determined, the resistance mechanisms should next be characterized. A rapid and simple bioassay in combination with synergists could be used to detect some metabolic resistance mechanisms. Synergists serve as enzyme inhibitors of metabolic detoxification enzymes such as esterases, P450s, and GSTs (Table 8). Bioassays incorporating synergists have been used widely to detect the role of different resistance mechanisms in many insect pests. For example, synergists, such as PBO, have been incorporated in the control of bed bugs, and to detect potential resistance mechanisms [127–129, 136]. However, not all resistance mechanisms could be characterized using synergists. Therefore, biochemical assays and molecular assays must be employed along with insecticide bioassays to detect the specific resistance mechanisms (Tables 6, and 9).

**Table 8:** Synergists as inhibitors of major metabolic detoxification enzymes

Insecticide detoxification enzymes	Synergists/inhibitors^a^
Cytochrome P450 monooxygenases (P450s)	PBO, sesamex
Esterases	EN16/5–1, DEF, TPP, IBP, K-1, K-2, sesamex, and SV-1
Glutathione S-transferases (GSTs)	DEM, EA and CF

*Abbreviations*: *DEF* S.S.S-tributlyphosphorotrithioate, *TPP* triphenyl phosphate, *IBP* S-benzyl diisopropyl phosphorothiolate, *K-1* (2-phenyl-4H-1,3,2-benzodioxaphosphorothiolate), *K-2* 2-phenoxy-4H-1,3,2-benzodioxaphosphorin 2-oxide, *sesamex* 5-[1-[2-(2-ethoxyethoxy) ethoxy]ethoxy]-1,3-benzodioxole, *SV-1*, O,O-diethyl-O-phenyl phosphorothiolate, *DEM* diethyl maleate, *EA* ethacrynic acid, CF chlorfenethol

^a^Data sourced from Brogdon & Chan [286], Heong et al. [147] and Lilly et al. [127]

**Table 9:** Published molecular markers of genes putatively involved in resistance mechanisms of bed bugs (*Cimex* spp.)

Resistance mechanism	Gene	Molecular assay	Primer sequence (5′˗3′)	Reference
*kdr*	V419 L	PCR and sequencing	F: AACCTGGATATACATGCCTTCAAGG; R: TGATGGAGATTTTGCCACTGATG	[54, 157]
	L925; I936F	PCR and sequencing	F: GGAATTGAAGCTGCCATGAAGTTG; R: TGCCTATTCTGTCGAAAGCCTCAG	[54, 157]
	V419L	AS-PCR	F(V): ATTCCTGGGATCATTCTACCTCg; F(L): ATTCCTGGGATCATTCTACCTCc; R: TGATGGAGATTTTGCCACTGATG	[90, 157]
	L925I	AS-PCR	F(L): ATTATGGGCAGAACAGTGGGTGCCc; F(I): ATTATGGGCAGAACAGTGGGTGCCa; R: TGCCTATTCTGTCGAAAGCCTCAG	[90, 157]
	V419L	QS	F: GTCCGTGGCACATGTTGTTCTTCA; R: CTGATGGAGATTTTGCCACTGATGC; R: CCTCTTCAGCAGCTTCTTCTTCTTC (for sequencing)	[160, 162]
	L925I	QS	F: GGTCTATCAGTTTTGAGGTCATTCAG; R: GGAGTTCGCCATCAGGGAATCTAT; F: GTGTTTAAGCTGGCTAAGTCATGGCC (For sequencing)	[160, 162]
	M918I; L1014F	PCR and sequencing	F: GGAATTGAAGCTGCCATGAAGTTG; R: TGCCTATTCTGTCGAAAGCCTCAG	[17]
Penetration resistance	Contig_1766 (CDA)	qRT-PCR	F: TGAATGCTATAAGAATCGTA; R: ATTACCAATACACCAACAA	[92]
	Contig_1762 (CHS)	qRT-PCR	F: TAATGAAGCAAGGCACTA; R: AATACTCCACACGATACC	[92]
	Contig_48951 (CPAP)	qRT-PCR	F: GTCCTCAGCACCAATCGT; R: GTTGTTGGAACTGTTGTTGATG	[92]
	Contig_17694 (LCP)	qRT-PCR	F: GCCACTACTATAACAGAG; R: ATTACCTCCAAGATTGAAT	[92]
	Contig_21630 (PCP)	qRT-PCR	F: CCAGATAATTCAAGAGATG; R: AGTCTAATCGGTCTATATG	[92]
	Contig_24229	qRT-PCR	F: CGCCAGGGCCGAGGAGTATG; R: ACGGGGTCGGCGGTGTAGTCT	[94]
	Contig_24231	qRT-PCR	F: CGACGATCATCCCCAATACAGTTT; R: AGGGGCGGCTTTAGCGACCACA	[94]
	Contig_24227	qRT-PCR	F: TTTCTTTTGGCAGCTTTGGTTGTA; R: CCTGCTTTCGGTCTGGGATTTG	[94]
	Contig_24230	qRT-PCR	F: GACTACTACGCCCACCCGAAATAC; R: GTGAGCAGCGTGGCCAGTCTTGTG	[94]
	Contig_24228	qRT-PCR	F: TCCCGCTGTTACCAAGACTCAATG; R: GCCAAAGCGACTGCAGGTGTATC	[94]
	Contig_2034	qRT-PCR	F: TACCGTTAATGCTGCTACACCAA; R: TCCCGAGGCGACGAAACCACT	[94]
	Contig_1629	qRT-PCR	F: AGGCCAGTCCAACACAACCAAC; R: TGCTGCCGTCCTCATTCTCC	[94]
	Contig_3037	qRT-PCR	F: ACGGCAGGATGGTCGAAGATTATG; R: AGGACGAGGGGCGGGCTGTGGT	[94]
	Contig_2322	qRT-PCR	F: CGAACCTGCCGGAAGTGACATAAA; R: TTGCGGCTGGTAGTACTGAGGTTG	[94]
	Contig_773	qRT-PCR	F: GCCGTTGAGCAGCAGCGATAA;	[94]
			R: CGTGGGGCGGAAGAAGGATT	
	Contig_2220	qRT-PCR	F: ATGGCACCAGGAGGGGAACTTA; R: GGTACTGGGGCTGGGCTCTGT	[94]
	Contig_1833	qRT-PCR	F: ACAATTCGGTGGTGCCCCTTTCT; R: TCGGCGACGTAGCTGACCTGGAC	[94]
	Contig_820	qRT-PCR	F: ATCAGCAGCCAAGTCGTAGGAAGC; R: GGAGGGGTTGGGAGGTGGTCT	[94]
	Contig_934	qRT-PCR	F: ACACCACTCCCGTTCCCATCGTC; R: CGTTCTCGTCGGCGGTGTAAGTCA	[94]
	Contig_2492	qRT-PCR	F: CGGTATCTCGGCGAAGGAACAG; R: GCGGCGTAGGGAGATGAGCA	[94]
	Contig_22513	qRT-PCR	F: CGACGGAACATACAACTGGGAATA; R: GTCGGCGGTGTAGGTCAACTGGAT	[90]
	Contig_15313	qRT-PCR	F: ACGGAACCCATCCCGATCCTTAAA; R: ATGCCGTTACCGGTTTCGTATTCC	[90]
	Contig_02621	qRT-PCR	F: ACTCCTGAAGTCCAAGCAGCAAGA; R: TCGGAAGGGTTGTCCAAATCGTGA	[90]
	Contig_8158	qRT-PCR	F: CCAAGCGGTCAAAGCAGCACATTT; R: AGCCTAGCGAGAGCTTGGTTGTAA	[90]
Metabolic resistance [1] P450s	CYP9 (Contig No.: N/A)	qRT-PCR	N/A	[104]
	CYP6DM2 (Contig No.: N/A)	qRT-PCR	F: CCCCCTTATGCTACCCGTTTGA; R: TTCGTCCTTTTTATGTCCGTCTGC	[113]
	CYP397A1 (Contig No.: N/A)	qRT-PCR	F: CTCGGGCTCACCACTCTCAACA; R: ACCGTCATGGCTCCCGTCAG	[113]
	CYP400A1 (Contig No.: N/A)	qRT-PCR	F: CCTGCGCGTTCGGAGTCAATA; R: CATCGGCTAAATAGAGGAAAAAGT	[113]
	Contig_19601 (CYP397A1V2)	qRT-PCR	F: TCGGAGGAATGGAAGAAG; R: CGTCATGGTATGGATGGT	[92]
	Contig_103 (CYP6A2)	qRT-PCR	F: AAGTTGTCCTAGAGTGTT; R: GAGATATGCGTGAATGTC	[92]
	Contig_22399 (CYP6A13)	qRT-PCR	F: CGTCATGGTATGGATGGT; R: TCGGAGGAATGGAAGAAG	[92]
	Contig_11345 (CYP397A1)	qRT-PCR	F: TATTGGAGTCGACAGGGCGTGAAA; R: TGACATCGCCCAATTGCTTGTAGC	[90]
	Contig_03764 (CYP398A1)	qRT-PCR	F: TGTCGACCCAATGATGGCTCTGAA; R: GAAATTGGAGGCCGATTTGGCGAT	[90]
	Contig_04490 (CYP6DN1)	qRT-PCR	F: GCGAGTCTGGGAAATTGTGCATGAAT; R: AATGCCCGATTACGATGTCAGGGA	[90]
	Contig_04099 (CYP4CM1)	qRT-PCR	F: ATTGGTAACATTGGAGGCCCTGGA; R: AGAGATTTGCCTTACCACCAGCGA	[90]
	ClCPR (Contig No. N/A)	qRT-PCR	F: TATGCCGCAGAATACGGACAACTC; R: ACCTGCAAATTCTTCACCAGTGCC	[122]
[2] Esterases	CE3959 (Contig No. N/A)	qRT-PCR	F: ACGTCTGGAGAAGGGCAACTGAAA; R: GACGGCCGGGTAGATGAAAACAAC	[90, 113]
	CE21331 (Contig_03262)	qRT-PCR	F: TCTCACGGGGACGAACTGCCTTAT; R: CCTGGTCTTCTGGGTATTTCTTCA	[90, 113]
[3] GSTs	gsts 1 (Contig No. N/A)	qRT-PCR	F: AGGAGAGCCAGTTAGATTTATGTT; R: AAGCGATTCCCACCGATTTT	[113]
[4] ABC- Transporters	Contig_1346	qRT-PCR	F: TGCTCTACATAATTCTGACAT; R: GTAGGACGGTATGAGGTA	[92]
	Contig_08506 (Abc8)	qRT-PCR	F: ATCCTGATGGGCCGAGTAAACCAT; R: TTCTGGAGGTGACCGTCAAGTTGT	[90]
	Contig_02154 (Abc9)	qRT-PCR	F: TTTAGCAACCGATGTGACGCAAGC; R: TGACCCAGACGTTGTCAACACAGA	[90]
	Contig_05955 (Abc10)	qRT-PCR	F: TCACAGCGGTCTTCCTGGATTCTT; R: AACTTCTGCGCGCACATTAGAACG	[90]
	Contig_09403 (Abc11)	qRT-PCR	F: ATGCAGCTCAGTAGGGTCGTCTTT; R: CGGGCCAAAGTCAAATCAGCACAT	[90]

*Abbreviation N/A* Not applicable, *AS-PCR* Allele-Specific PCR, *QS* Quantitative Sequencing *qRT-PCR* quantitative Real-Time PCR, *F* Forward primer, *R* Reverse primer

### Biochemical assays

Biochemical assays use model substrates to detect elevated activity of metabolic enzymes involved in insecticide resistance in individual insects. Over the last two decades, biochemical assays have been used successfully to detect and monitor insecticide resistance in numerous insects [203] especially in combination with insecticide bioassay. Karunaratne et al. [56] surveyed insecticide resistance and potential resistance mechanisms in Sri Lankan *C. hemipterus* based on toxicity bioassays [36, 39] and biochemical assays [203]. They found that *C. hemipterus* showed high levels of resistance to DDT and malathion, and detected elevated levels of GSTs and esterases as well. Yoon et al. [156] used biochemical assays to identify resistance mechanisms responsible for deltamethrin resistance in a New York *C. lectularius* strain, although there were no differences in the activity of the enzymes evaluated. Adelman et al. [113] also used biochemical assays to detect the differential activity of detoxification enzymes, which suggested that metabolic resistance probably was associated with pyrethroid resistance in *C. lectularius*. Romero & Anderson [64] evaluated the activities of metabolic detoxification enzymes (P450s, GSTs, and esterases) in *C. lectularius* using biochemical assays. They found that metabolic resistance is probably involved in resistance to neonicotinoids. However, it is important to clarify that the presence of elevated levels of enzymes alone is not a direct evidence to demonstrate their involvements as resistance mechanisms, unless it could be shown through in vivo metabolism and/or synergism studies that these enzymes were involved [105].

### Molecular assays

Detection of insecticide resistance using molecular assays could provide early warning of the development of insecticide resistance and the specific resistance mechanism, which would allow the choice of insecticides to be decided more accurately. For example, *kdr*-resistance mutations could be identified in many DDT- and/or pyrethroid-resistant insects. Multiple molecular assays have been developed to detect *kdr* mutations, such as direct DNA sequencing analysis, allele-specific PCR (AS-PCR), Heated Oligonucleotide Ligation Assay (HOLA), Sequence Specific Oligonucleotide Probe Enzyme-linked ImmunoSorbent Assay (SSOP-ELISA), PCR-Dot Blot, Fluorescence Resonance Energy Transfer/Melt Curve Analysis (FRET/MCA), High Resolution Melt (HRM), and TaqMan Real-Time PCR assays, in a range of insect pests [204], including bed bugs (Table 9) [17, 54, 90, 92, 113, 156–158, 162]. Direct DNA sequencing analysis, including QS (Quantitative Sequencing) [160, 162] is the most common molecular assay used to identify *kdr* mutations (e.g. V419L, L925I) in field-collected bed bug strains (Table 9) [54, 92, 113, 157, 158]. However, this analysis is not practical for studying large-scale populations. Other assays such as AS-PCR [90, 157] can be used to detect *kdr* mutations in field-collected strains (Table 9). As noted by Bass et al. [204], a more sensitive and specific molecular assay (e.g. TaqMan Real-Time PCR assay) should be developed to identify *kdr* mutations.

The recent transcriptome and genomic studies in bed bug populations have revealed that multiple candidate genes that putatively mediate resistance mechanisms may be present in bed bug populations (Table 1) [90, 92, 93, 113, 197]. In these studies, overexpression of genes that encode metabolic enzymes (e.g. P450s, esterases, and GSTs) and genes that encode cuticular proteins for thickening or remodelling cuticle to reduce the insecticide penetration rate, were used as discriminating criteria to identify genes that are likely associated with insecticide resistance, especially with pyrethroid resistance, in bed bugs [90, 92, 94, 104, 113], although they need to be further experimentally validated. Based on the quantitative RT-PCR technology, many molecular markers have been developed to monitor those putative resistance-associated genes based on these studies (Table 9) [90, 92]. Additionally, RNAi techniques also provides a promising approach to further validate the gene(s) that governs resistance in bed bugs [90, 122]. Lastly, other approaches, such as transgenic expression [143] and metabolism studies [105], can be conducted to directly validate the gene(s) governing resistance in bed bugs.

## Conclusions

Over the last two decades, bed bugs have undergone a major resurgence around the world. The widespread presence of insecticide resistance in field bed bug populations may be the single most important factor responsible for the bed bug resurgence. Transcriptomic and genomic studies have significantly revolutionized insecticide resistance research in bed bugs over recent years. Multiple physiological-based mechanisms are putatively associated with bed bug insecticide resistance. These mechanisms include reduced penetration by thickening or remodelling cuticle (e.g. upregulation of cuticular-related protein genes), metabolic resistance (e.g. increased metabolic activities of detoxification enzymes), and target site insensitivity (*kdr* mutations). However, the involvement of most of the candidate genes associated with insecticide resistance found through transcriptomic and genomic approaches still need to be verified by empirical functional approaches such as RNAi, gene functional characterization, metabolism/biotransformation studies, and neurophysiological studies. The progress in understanding insecticide resistance mechanisms mentioned herein is mainly focused on the common bed bug *C. lectularius*. Little is known on insecticide resistance mechanisms in the tropical bed bug *C. hemipterus*, although it is likely to share similar mechanisms. Bioassay methods are relatively simple to perform and provide standardized data to monitor insecticide resistance. However, they have several practical limitations. For instance, bioassays require a susceptible strain for comparison and a large quantity of insects for testing. Ideally, biochemical, molecular assays and insecticide bioassays could be concertedly performed to detect insecticide resistance and its mechanisms. The latter two assays have advantages, such as requiring a smaller sample size, and could accurate identify the gene(s) that is/are associated with the resistance mechanisms. However, these assays require sophisticated and costly equipment and reagents to perform which may not be readily available in developing countries. In addition, it would be counter-productive to use biochemical and molecular monitoring assays as a stand-alone approach without empirical validation of resistance status.

## Abbreviations

ABC-transporters: ATP-binding cassette transporters
AChEs: Acetylcholinesterases
ADP: Adenosine diphosphate
ATP: Adenosine triphosphate
BHC: Benzene hexachloride
CDA: Chitin deacetylase
CF: Chlorfenethol
CHS: Chitin synthase
CIN-1: A *Cimex lectularius* strain collected in 2005 in Cincinnati, OH
CPAP: Cuticular protein analogous to peritrophin
CPR: NADPH-Cytochrome P450 Reductase
CPRR: Cuticular protein with the rebers and riddiford consensus
DDT: Dichloro-diphenyl trichloroethane
DDVP: Dichlorvos
DEF: *S.S.S*-tributlyphosphorotrithioate
DEM: Diethyl maleate
EA: Ethacrynic acid
EN16/5–1: 6-[2-(2-butoxyethoxy) ethoxymethyl]-5-propyl-2, 3-dihydrobenzofuranby
FCVB: Filter Contact Vial Bioassay
FL-BB: A susceptible *C. lectularius* strain collected from Gainesville, Florida
FRET/MCA: Fluorescence Resonance Energy Transfer/Melt Curve Analysis
GABA receptor: γ-aminobutyric acid receptor
*gamma*-HCH: *gamma*-hexachlorocyclohexane
GSH: Reduced glutathione
GSTs: Glutathione *S*-transferases
HOLA: Heated Oligonucleotide Ligation Assay
HRM: High Resolution Melt
IBP: S-benzyl diisopropyl phosphorothiolate
IRAC: Insecticide Resistance Action Committee
IRM: Insecticide resistance management
K-1: (2-phenyl-4H-1,3,2-benzodioxaphosphorothiolate)
K-2: 2-phenoxy-4H-1,3,2-benzodioxaphosphorin 2-oxide
*kdr*: knockdown resistance
LA-1: A susceptible *C. lectularius* strain collected in 2006 in Los Angeles, CA
LCP: Larval cuticle protein
METI: Mitochondrial electron transport inhibitors
nAChRs: Nicotinic acetylcholine receptors
NADPH: Nicotinamide Adenine Dinucleotide Phosphate
OCs: Organochlorines
OPs: Organophosphates
P450s: Cytochrome P450 monooxygenases
PBH: 3-Phenoxybenzyl hexanoate, a surrogate substrate for carboxylesterases and oxidases
PBO: Piperonyl butoxide
PCP: Pupal cuticle protein
qRT-PCR: Quantitative Real-Time PCR
QS: Quantitative Sequencing
RACE: Rapid amplification of cDNA ends
RNAi: dsRNA-mediated interference
SEM: Scanning electron microscope
sesame: 5-[1-[2-(2-ethoxyethoxy) ethoxy]ethoxy]-1,3-benzodioxole
SSOP-ELISA: Sequence Specific Oligonucleotide Probe Enzyme-linked Immuno Sorbent Assay
SV-1: O,O-diethyl-O-phenyl phosphorothiolate
TPP: Triphenyl phosphate
VGSC: Voltage-gated sodium channel
WOR-1: a *C. lectularius* strain collected in 2007 in Worcester, MA
